# Living the good life? A systematic review of behavioural signs of affective state in the domestic horse (*Equus caballus*) and factors relating to quality of life. Part I: Fulfilment of species-specific needs

**DOI:** 10.1017/awf.2024.38

**Published:** 2024-10-21

**Authors:** Carol Hall, Rachel Kay

**Affiliations:** 1 School of Animal, Rural and Environmental Sciences, Nottingham Trent University, Southwell, Nottinghamshire NG25 0QF, UK; 2 National Equine Welfare Council, Slad Lane, Princes Risborough, Bucks HP27 0PP, UK

**Keywords:** Animal welfare, equine ethogram, Five Domains model, human behaviour change, social licence, social needs

## Abstract

The welfare of horses involved in sport and leisure activities has come under increasing scrutiny, both from within the equine sector and from the public. A systematic review of scientific evidence was conducted to derive observable, evidence-based behavioural measures of positive or negative affective state and factors relating to quality of life in the domestic horse (*Equus caballus*). Two separate searches (including the terms ‘emotion/affect’, or ‘stress’) were conducted, with 179 articles being retained. In Part I of this two-part review (companion paper published simultaneously), articles relating to the extent to which the species-specific needs of the horse are fulfilled in the home environment (n = 21), in relation to social grouping (n = 20), and during weaning (n = 14) were reviewed. Experimental tests of affective state in relation to housing and management (n = 8), and studies relating to stereotypical behaviour (n = 7) were also included. Opportunities for behavioural expression were dependent upon the provision of social and physical resources. Withdrawn or agitated behaviours *in situ*, avoidance behaviour during handling and agonistic intra-species interactions were indicative of negative affective state. Affiliative intra-specific social interactions, and forage ingestion were considered positive signs. For horses to live a good life, their need for space, companionship, and forage must be prioritised from birth and throughout their lives.

## Introduction

Ethical and welfare issues have been widely identified and reported on in relation to many aspects of equestrianism, including training and competition practices, general management, and horse breeding (McLean & McGreevy 2010; Campbell 2017; Condon *et al.* 2022; McManus 2022). This has led to concerns regarding the inclusion of horses in both sporting (Campbell 2016) and leisure activities (Horseman *et al.* 2016). Under increasing public scrutiny, the equestrian community urgently needs to demonstrate that it is accountable for the welfare of horses throughout all stages of their lives (McManus 2022; Heleski 2023), and that it is actively working to improve their quality of life (Heleski 2023). Failure to do so may result in equestrian sports’ Social Licence to Operate (SLO), an unwritten social contract between society and participants in equestrian sports, being withdrawn (McManus 2022; Waran 2023). If this happens, equestrian activities may subsequently become subject to external regulation, or potentially be banned altogether (McManus 2022; Waran 2023).

Scientific thinking regarding what constitutes good animal welfare has advanced significantly over recent decades. There is now widespread acceptance that it is the animal’s perception of its physical and emotional state that matters (Webster 2013), and that for an animal to live a good life it must be provided with opportunities for positive experiences, not just protected from negative ones. The term affective state is used to describe emotions and other feelings associated with brain states induced by sensory input and cognitive processes that are experienced as either pleasant or unpleasant (Kirkwood 2007; Fraser 2008). An animal’s quality of life (QOL) is reliant upon the balance between positive (pleasant) and negative (unpleasant) experiences over time, which largely determines how valuable each animal’s life is for that individual animal from the animal’s point of view (Yeates 2016). Throughout this review, welfare and QOL will be considered as synonymous, but to avoid the potential negative connotations commonly associated with the term welfare, and to facilitate a focus on providing positive experiences, QOL is considered the preferable term for future applications (Furtado *et al.* 2021).

To enable stakeholders and researchers to accurately assess the QOL of a horse throughout its lifetime, evidence-based measures that are relevant and feasible for use across the equine sector, are required. The first stage in developing a framework upon which to base such an assessment involves identifying behaviour indicative of affective state, with the proviso that individual differences in behavioural expression will occur (Manrique *et al.* 2021). Subsequently, factors found to influence behavioural expression and, by implication, affective state, can be adapted to facilitate improvements in horse QOL which, in turn, can be measured through behavioural change.

To enable the assessment of both physical and mental well-being, two main questions were posed by Dawkins (2004): is the animal healthy and does it have what it wants? Although physical health can to a certain extent be measured, assessing whether an animal has what it wants is more of a challenge, particularly in a ‘traditionally’ managed domestic species such as the horse. A comparison with the behaviour of feral horses highlights the extent to which this management generally restricts the natural behavioural repertoire of the horse (Waran 1997) and suggests that most do not have what they would ideally want. A study by Schatzmann (1998) demonstrated that sport horses at winter pasture in Switzerland, prioritised being in the company of, or within view of, other horses and that regardless of weather conditions they preferred eating forage outside rather than in individual indoor boxes. These findings support the conclusion that what horses want are friends, forage, and freedom (Fraser 2012). To derive an answer to this second question posed by Dawkins (2004), it is necessary to include information about what resources are available to the animal and how the animal behaves in response to this provision.

Multiple factors contribute to an animal’s affective state, and these are encapsulated in the Five Domains model developed by Mellor *et al.* (2020). The model was initially modified to facilitate the identification of experiences that an animal may have that relate to positive affect and the potential to enhance welfare (Mellor & Beausoleil 2015), and subsequently to revise the domain of ‘behaviour’ to refer to behavioural interactions an animal has with other animals, humans, and the environment (Mellor *et al.* 2020). An important adaptation to this model, which aligns with the question of whether the animal has what it wants (Dawkins 2004), was incorporated in the guiding principles for humane livestock farming in The Netherlands (Council on Animal Affairs 2021). As well as the need to provide good nutrition, housing, and health, these include the need to provide the animal with sufficient opportunities to fulfil its natural behavioural needs. Only if these requirements are met can the animal experience a positive mental state (Council on Animal Affairs 2021). This latter principle is particularly pertinent when considering the QOL of the domestic horse (*Equus caballus*).

The application of the Five Domains model to assess the quality of life of any animal requires species-specific knowledge of both the behaviour and the behavioural needs of that species. Deriving conclusions about the affective state of the animal based on the other four domains, is undoubtedly challenging, and the association between behaviour and subjective experience unclear. Characterisation of animal emotion in terms of the two components of arousal and valence (Mendl *et al.* 2010) provides a basis for assessing affective state. As noted by Hall *et al.* (2018), both behavioural and physiological measures yield information relating to arousal, but often conflicting evidence in relation to valence. Comprehensive lists of horse behaviour with descriptors have been compiled by McDonnell (2003) and Waring (2003), as well as ethograms identifying specific behaviours associated with negative affective state, including signs of stress (for example, Young *et al.* 2012) and equine discomfort (Torcivia & McDonnell 2021). Despite the move towards providing positive experiences and potentially positive affective state (Mellor & Beausoleil 2015), conclusions regarding behavioural signs of positive affective state in the horse are generally based on the absence of negative signs. Such conclusions may not be warranted.

Identifying behavioural signs of affective state is key to promoting practices that provide the horse with positive experiences, and therefore a good QOL. A systematic review of scientific evidence was conducted to derive observable, evidence-based, behavioural measures of positive or negative affective state and factors that relate to QOL in the domestic horse. Features of management and training that may compromise or improve QOL will be identified. Those relating to the fulfilment of species-specific needs are presented in Part I of this review, those relating to the horse-human relationship in Part II (a companion paper published simultaneously; Hall & Kay 2024). The findings of Parts I and II will be combined in Part II to identify the changes needed in management and training to achieve a good life for all horses.

## Materials and methods

### Search strategy

The PRISMA 2020 statement was used as guidance in the collection and presentation of the empirical evidence referred to in this review (Page *et al.* 2021). During February 2023, a systematic literature search was performed to identify evidence-based behavioural indicators of equine affective (emotional) state and associated factors. The electronic databases searched were Science Direct, PubMed, Scopus, Web of Science and PubPsych. To ensure the potential inclusion of evidence of behaviour indicative of both negative and positive affect, two separate search queries were specified, one with the terms emotion/affect, and a second with the term stress:

- Search 1: ‘(emotion* OR affect* OR cognit*) AND (behav* OR welfare) AND (horse OR pony OR equine)’
- Search 2: ‘(stress AND behav*) AND (horse OR pony OR equine)’

Where possible, the searches were filtered for research articles only, and the inclusion of the search terms in the title, abstract and keywords of the article. Filters for individual databases varied slightly, with restriction to ‘Title, Abstract, and Keywords’ only available in Science Direct, PubMed and Scopus. In the Web of Science, the restriction to ‘Abstracts’ was used. In Science Direct the use of * (referred to as a wildcard) was not permitted and the search terms used were revised accordingly (in Search 1 to ‘[emotion OR affective OR cognition] AND [behaviour OR welfare] AND [horse OR pony OR equine]’ and in Search 2 to ‘[stress AND behaviour] AND [horse OR pony OR equine]’). In PubPsych, no filters were used, and the search terms were simplified to ‘horse AND emotion’ and ‘horse AND stress AND behaviour.’

### Inclusion criteria

A preliminary assessment of the relevance of the articles was carried out for each search. All peer-reviewed experimental and observational studies (primary research papers only) referring to equine emotional responses, equine stress, and emotional state (horses and/or ponies only) were included in the initial pool. No criteria relating to the date of publication were used in the searches. Undergraduate dissertations, post-graduate theses, textbooks, conference proceedings, review papers and publications not in the English language were excluded. Studies published both in abstract and full paper form were only evaluated in their full paper format. For each database, the total number of articles initially identified by each search was recorded. Within search duplicates (from different search engines) were removed at this first screening stage. The number of articles identified per year was calculated for each search separately to inform any change in article focus (Search 1 focused on emotion and Search 2 on stress).

The remaining articles were downloaded to Mendeley desktop. The articles were then assessed and those fulfilling the following criteria were retained. All studies were required to include a subject number greater than four, in any environment, either be observational or include a clear description of methods used, and needed to provide evidence to support the attribution of emotional state. Studies based upon quantitative and qualitative data were included. Articles that did not provide details of the methods used and/or justification for associations between emotional state and behaviour, were excluded.

### Data extracted

For each article that fulfilled the inclusion criteria the following information was extracted: the scenario involved (experimental and observational), the behaviour recorded (including the type of ethogram used and cited sources), the subjective experience attributed to the behaviour (positive/negative valence, emotional response, affective state, stress), the nature of the supporting evidence for this conclusion (reference to past studies, physiological measures, pain/pain-inducing procedures, circumstances typically associated with positive or negative experiences, affiliative/agonistic social interactions, approach/avoidance behaviour, evidence of choice/preference) and factors that affected the behaviour expressed (including sex, age, social and environmental features). Features of study design were recorded and reported in relation to each scenario. For each retained article the following details were recorded: the focus of the study, study design and duration, subject numbers, age and sex, the type of behaviour recorded, the type of ethogram used, and the type of supporting evidence presented. These details are available online in the Supplementary material for this study (Part I) and Part II (Hall & Kay 2024).

## Results and Discussion

**Figure 1.**
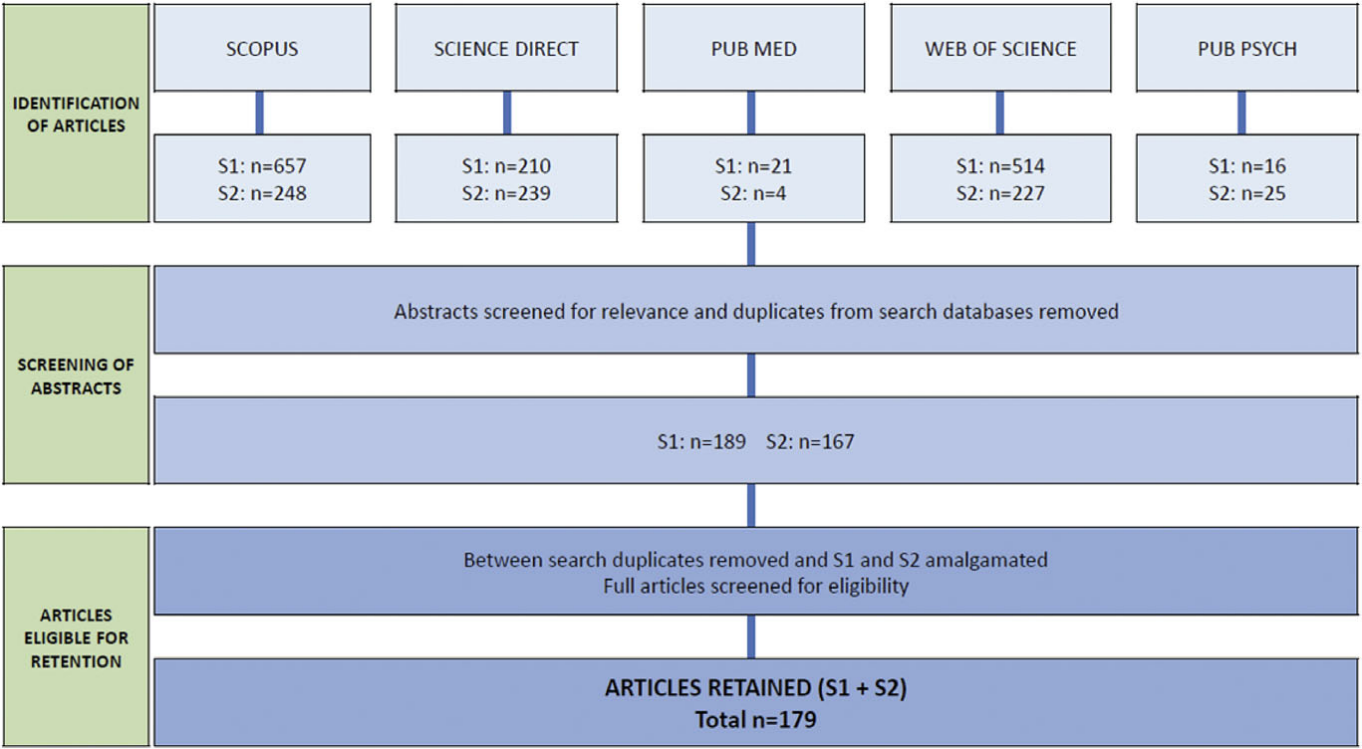
Systematic literature search procedure, including the number of articles identified at each stage of the review, and the total number of articles retained.

The total number of articles identified by each search database for Search 1 and Search 2, the number of articles retained following the initial screening of abstracts for relevance (species, behaviour recorded and related to affective state) and duplication (between databases), and the final number of retained articles eligible for inclusion in this review (Search 1 and 2 combined) are shown in Figure 1. A total of 179 articles were retained.

**Figure 2.**
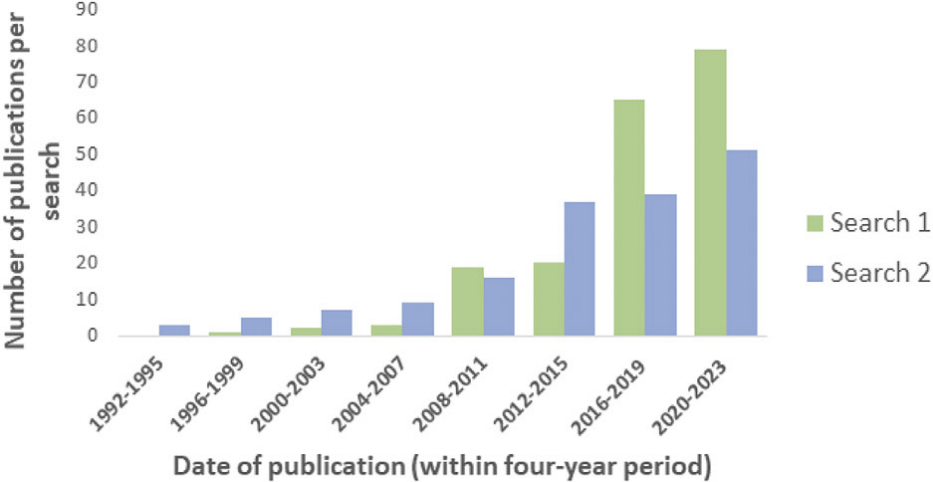
Number of publications identified under each search term (Search 1 and Search 2) for each four-year period (the period 2020–2023 included three years and two months only).

Publications identified per four-year period for each search are shown in Figure 2 (the most recent period consists of only three years and two months). No articles pre-1992 were identified. The number of publications per year has increased overall, with numbers identified in Search 1 overtaking those identified in Search 2 from 2016 onwards.

The change in emphasis on the promotion of positive states to enhance well-being rather than purely the avoidance of negative states (as was previously the focus of many animal welfare studies) was reflected in the comparative number of articles identified using the two search terms (emotion/affect or stress). The Five Domains model, as used to assess animal welfare, was extended to incorporate positive welfare states in the article by Mellor and Beausoleil (2015). This changing approach is reflected in the post-2015 relative increase in articles identified in Search 1 (emotion/affect), as illustrated in Figure 2.

The study scenarios relating to the fulfilment of species-specific needs included in the retained articles (n = 70), and the number of articles per scenario are shown in Table 1. The scenarios included in Part I of this review were selected for their association with the extent to which the species-specific needs of the horse were fulfilled by management in the home environment, in relation to social grouping, and during weaning. All the experimental tests of affective state involved an assessment of the effect of housing and management conditions on judgement bias and were consequently included in Part I, as were all studies relating to stereotypical behaviour, the development of which is generally associated with a failure to provide for species-specific needs (Sarrafchi & Blokhuis 2013). The behaviour recorded in these scenarios was not influenced by interactions with humans, except to the extent that the latter determined the features of management under review. The behaviour of horses in the many and varied situations where more direct interactions with humans occurs, including those where human interpretation of horse behaviour should guide subsequent interactions, is reviewed in Part II (Hall & Kay 2024).

**Table 1.** Study scenarios relating to the fulfilment of species-specific needs identified in the literature search, the related table in the Supplementary material and the number of articles retained per scenario. The scenarios are listed in descending order according to the number of articles retained

Type of study scenario	Table number in Supplementary material	Number of articles retained
Home environment	S1.1	21
Intra–species social behaviour	S1.2	20
Weaning	S1.3	14
Experimental tests of affective state	S1.4	8
Stereotypical behaviour	S1.5	7

The scenarios are listed in descending order according to the number of articles retained. Study scenarios relating more specifically to interactions with humans are reported in Part II (Hall & Kay 2024) of this review. The behaviour attributed to affective state and the factors affecting this behaviour are subsequently reported for each separate scenario. In addition, generic factors found to relate to behavioural expressions of affective state, and behavioural characteristics associated with specific affective states, are evaluated below. Further details of the experimental design used in the articles retained under each scenario are available in the Supplementary material (Tables S1.1–S1.5).

### Studies reporting behaviour associated with affective state and related factors for each scenario

#### The home environment

The greatest number of articles retained in Part I of this review (21) focused on the behaviour of the horse in its home environment. The behaviour recorded varied according to the specific focus of the study and the features of the home environment that were under investigation. Features included stable type/size/design (Burla *et al.* 2017; Lesimple *et al.* 2019; Ruet *et al.* 2019; Marliani *et al.* 2021), paddock vs stabling/turn-out opportunities (Harewood & McGowan 2005; Werhahn *et al.* 2012; Pessoa *et al.* 2016; Suagee-Bedore *et al.* 2021), the social environment (individual, paired or group housing; Harewood & McGowan 2005; Visser *et al.* 2008; Werhahn *et al.* 2012; Yarnell *et al.* 2015), bedding area/depth (Burla *et al.* 2017; Greening *et al.* 2021), mode of forage provision (Melvin *et al.* 2020; Sundman *et al.* 2022; Baumgartner *et al.* 2023) and environmental enrichment (Lansade *et al.* 2014, 2022).

The retained articles used one of two main approaches, either assessing the impact of current (long-term) housing or testing the effect of specific conditions set up experimentally for shorter periods. In five of the retained articles the effect of long-term housing type and the association between housing features and behavioural and physiological signs of welfare was assessed (Lesimple *et al.* 2019; Ribeiro *et al.* 2019; Ruet *et al.* 2019; Sénèque *et al.* 2019; Sauveroche *et al.* 2020). Where a timescale for the current housing was provided, this ranged from > 30 days (Ribeiro *et al.* 2019) to > 9 months (Ruet *et al.* 2019). The impact of shorter-term exposure to differing housing situations was assessed either by comparing different horses kept under different conditions for a specific length of time (between subjects: 12 weeks; Visser *et al.* 2008, five weeks; Lansade *et al.* 2014), or by repeated measures where all horses underwent all conditions each for a set time-period which varied from 1 h (Suagee-Bedore *et al.* 2021), 24 h (Harewood & McGowan 2005), five days (Yarnell *et al.* 2015), six days (Greening *et al.* 2021), eleven days (Burla *et al.* 2017), 14 days (Werhahn *et al.* 2012; Melvin *et al.* 2020), 16 days (Pessoa *et al.* 2016), to approximately 34 days non-consecutively in experiment 2 of the study by Lesimple *et al.* (2019). Periods of adaptation to each condition were generally included, with behavioural and physiological assessments taking place towards the end of the period in each condition (for example, Burla *et al.* 2017 recorded behaviour over the last three days of eleven-day treatment periods). Subject numbers included in the studies assessing long-term effects of housing ranged from 32 (experiment 1; Lesimple *et al.* 2019) to 187 where behaviour was recorded via scan sampling for 50 days over a nine-month period (Ruet *et al.* 2019). Subject numbers included in the shorter-term studies ranged from six (Harewood & McGowan 2005) to 38 (Burla *et al.* 2017). See Table S1.1 in the Supplementary material for details relating to all studies retained in this scenario.

#### Behaviour recorded

Behaviour in the home environment indicative of affective state (positive or negative) and the type of supporting evidence reported is shown in Table 2(a). The impact of different types of home environment was also assessed by means of additional tests, including learning trials (Lansade *et al.* 2014; Greening *et al.* 2021), handling tests (Harewood & McGowan 2005; Yarnell *et al.* 2015), reactivity tests (Visser *et al.* 2008) and ridden tests (Werhahn *et al.* 2012; Pessoa *et al.* 2016). Where the home environment was found to affect behaviour in these scenarios this is identified as a factor in the relevant section in Part II (Hall & Kay 2024) of this review.

**Table 2.** (a) Behaviour in the home environment indicative of affective state (positive or negative), supporting evidence*, and (b) factors affecting this behaviour

	Signs of positive affective state	Basis of justification	Signs of negative affective state	Basis of justification
**2(a) BEHAVIOUR IN THE HOME ENVIRONMENT**				
**Behavioural States**				
Standing	Increased standing calm ‘Alert’ posture (elevated neck with ears pricked forward, looking intensely at the environment)^a^	PS (Pessoa *et al.* 2016; Lesimple *et al.* 2019); PHYS (Sauveroche *et al.* 2020) PS (Ruet *et al.* 2019)	Increased standing alert / vigilant Increased standing dozing, ‘withdrawn’ posture (neck horizontal at same level as back, fixed stare, ears, and head static) ^b^ ‘Alert’ posture (elevated neck and ears pointed forward) Flat/hollow dorsal profile (neck/croups areas) Unresponsive	–ve SIT (Visser *et al.* 2008) –ve SIT (Ruet *et al.* 2019) –ve SIT (Lansade *et al.* 2014) PS (Sénèque *et al.* 2019) PS (Ruet *et al.* 2019, 2020)
Lying	Increased lying (ventral or lateral recumbency)	+ve SIT (Lansade *et al.* 2014) PS (Burla *et al.* 2017; Greening *et al.* 2021)	Decreased lying	PS, PHYS (Werhahn *et al.* 2012) PS (Burla *et al.* 2017; Greening *et al.* 2021)
Non–social activity	Decreased avoidance behaviour	APP (Melvin *et al.* 2020)	Agitated movements	PS (Harewood & McGowan 2005)
Social interaction (with horses)	Increased positive interactions Decreased agonistic threats Allogrooming / positive response to allogrooming^d^	+ve SOC, PHYS (Yarnell *et al.* 2015); PHYS (Sauveroche *et al.* 2020) PS, +ve SOC (Melvin *et al.* 2020) +ve SOC (Lansade *et al.* 2022)	Increased negative interactions Increased agonistic behaviour and low–level threats^c^ Decreased social interaction	PHYS, – ve SOC (Yarnell *et al.* 2015) PHYS, –ve SOC (Suagee–Bedore *et al.* 2021; Baumgartner *et al.* 2023) PS (Marliani *et al.* 2021)
Feeding	Increased feeding	PS (Visser *et al.* 2008; Yarnell *et al.* 2015; Lesimple *et al.* 2019) PHYS (Yarnell *et al.* 2015) +ve SIT (Lansade *et al.* 2014)	Decreased feeding	PS, PHYS (Yarnell *et al.* 2015)
Stereotypical behaviour			Development / display of stereotypical behaviour (locomotory and oral, head tossing)	PS (Visser *et al.* 2008; Yarnell *et al.* 2015; Lesimple *et al.* 2019; Ribeiro *et al.* 2019; Ruet *et al.* 2019, 2020; Marliani *et al.* 2021)
**Behavioural Events**				
Head / neck movements	Ears forwards, ears sideways	+ve SIT (Stomp *et al.* 2018a,b)	Ears backwards Sniffing, licking stable walls / bars Repetitive head swinging	–ve SIT (Lansade *et al.* 2014)
Leg / hoof movements			Increased pawing, stamping Repetitive pawing, kicking the wall	–ve SIT (Visser *et al.* 2008; Lesimple *et al.* 2019) –ve SIT (Lansade *et al.* 2014)
Vocalisations	Occurrence of snorts Decreased vocalisation (neighs)	+ve SIT (Stomp *et al*. 2018a,b) +ve SIT (Lansade *et al.* 2014)	Increased high pitched whinnies; increased neighing, snorting	–ve SIT (Harewood & McGowan 2005; Visser *et al.* 2008)
Interactions with humans			Aggression Increased tension shown by horse	–ve SOC (Ruet *et al.* 2019, 2020) PS, –ve SOC (Harewood & McGowan 2005; Yarnell *et al.* 2015)
**2(b) FACTORS AFFECTING BEHAVIOUR IN THE HOME ENVIRONMENT**				
Features of stabling	Area of bedding positively associated with time spent in recumbency, necessary for REM sleep Grills between stables allowing neighbouring horses some visual, tactile, and olfactory contact increased resting and foraging behaviour, and reduced stereotypical behaviour and vigilance. Having an open top door at the front of the stable (allowing horse to put its head out) increased agitation and stereotypical behaviour (particularly weaving). Horses housed in stables with a window to the outside and with straw bedding were found to be less aggressive to humans than those stabled without these features. Horses stabled in large stalls with visual and/or physical contact with other horses showed more exploratory behaviour (sniffing stimulus) in a novel object test, than those in more restrictive housing.		(Burla *et al.* 2017; Greening *et al.* 2021) (Lesimple *et al.* 2019) (Ruet *et al.* 2019) (Bulens *et al.* 2015)	
Forage provision	Inadequate forage provision and long gaps between feeds was associated with increased occurrence of undesirable behaviours (including stereotypies) in stabled horses. The provision of restricted food resources in group housed horses results in increased agonistic behaviour, which can be reduced by ensuring the number of access points far outweighs the number of horses. Increased agonistic threats and avoidance behaviour occurred in group housed horses fed forage from round bales within a ring feeder and small holed net, compared with large holed or no net. When forage was present horses were less inclined to explore the environment and showed less interest in novel objects, than when no food was available.		(Ribeiro *et al.* 2019) (Baumgartner *et al.* 2023) (Sundmann *et al*. 2022) (Bulens *et al.* 2015)	
Features of paddock	Preference for forage (hay) presented in enclosed feeders shown over open round bales in ring feeder, with fewer agonistic threats and avoidance behaviour associated with preferred feeder. Social interactions (both positive and negative) decrease as paddock size increases (see also Table 3[b]).		(Melvin *et al.* 2020) (Majecka & Klawe 2018)	
Time spent in stable / turnout	Horses with no access to turn–out (confined to stable when not in work) showed less signs of relaxation (increased time spent standing alert, reduced time spent standing relaxed) and fewer positive social interactions than when they were turned out into a paddock for 12 h daily. The longer horses spent stabled in individual boxes, the more likely they were to express unresponsiveness to the environment. When horses were not allowed any turn–out, they spent longer investigating the stable environment and less time lying down than when provided with turn–out periods in pairs. Increased size of turn–out area reduces occurrences of agonist behaviour (providing at least 342 m^2^ per horse may reduce the chance of injury in horses accustomed to group turn–out).		(Pessoa *et al.* 2016) (Ruet *et al.* 2019) (Werhahn *et al.* 2012) (Suagee–Bedore *et al.* 2021)	
Social grouping	Behavioural signs of stress and the development of stereotypical behaviours was more prevalent in two–year–old horses housed in individual stabling for the first time compared to those housed in pairs. Reduced time spent feeding was found when horses were stabled individually with no social contact, compared to paired or group housing and individual stabling that allowed some social contact by means of open bars between neighbouring stables.		(Visser *et al.* 2008) (Yarnell *et al.* 2015)	
Environmental enrichment (EE)	Provision of auto–brushes as EE stimulated allogrooming in a large group of horses. An enriched stable environment (large individual stables, straw bedding, daily turnout in groups, the provision of various olfactory, gustatory, and auditory enrichments) had a positive effect on the response of young horses to humans, sensory stimulation, signs of fear and learning performance that lasted for at least three months post EE.		(Lansade *et al.* 2022) (Lansade *et al.* 2014)	
Age of horse	When housed in a large group, older horses spent less time sternally recumbent and more time resting standing than younger horses.		(Hildebrandt *et al.* 2022)	

* Key to supporting evidence: Past studies (PS), physiological measures (PHYS), assumption of associated pain (PIP), situations deemed positive or negative (+ve/-ve SIT), positive or negative social interactions (horse / human) (+ve/-ve SOC), approach / avoidance (APP/AVO), choice (PREF).

^a^ *Postural attributes:* Elevated neck with ears pricked forward, looking intensely at the environment; ^b^ Neck horizontal at same level as back, fixed stare, ears, and head static.

c *Agonistic behaviours*: (chasing, contact biting, and kicking) and *low-level threats* (pinned ears, tail swishing, bite, and kick threats).

d *Positive response to allogrooming*: neck moderately raised, eyes open or half-closed, upper lip extended, ears turned backwards almost in line with the nose.

Ethograms were used in the 21 retained articles. One study (Stomp *et al*. 2018a) focused on a very specific behaviour (snorts), and there were only two examples where lists of behaviour were used without further descriptors (Werhahn *et al.* 2012 for the tests of ridden behaviours; Lansade *et al.* 2014: response to enrichment). Where the ethogram had been developed with reference to previous studies, a total of 30 different references were cited as sources, with only three being cited by more than one study (McDonnell & Haviland 1995; Cooper *et al.* 2000; McDonnell 2003 were each cited by two studies). The behaviours recorded varied according to the focus of the study. Activity patterns and behaviour *in situ* were used to assess the impact of housing type (Visser *et al.* 2008; Werhahn *et al.* 2012; Yarnell *et al.* 2015; Pessoa *et al.* 2016; Ribeiro *et al.* 2019; Ruet *et al.* 2019; Marliani *et al.* 2021), with studies assessing sleep patterns including detail of types of recumbency (Burla *et al.* 2017; Greening *et al.* 2021). Only behaviours identified in previous studies as being indicative of stress were recorded by Harewood and McGowan (2005), but no specific references were cited to support the ethogram used. Stereotypical and other abnormal repetitive behaviours were recorded by Lesimple *et al.* (2019), Ribeiro *et al.* (2019), Ruet *et al.* (2019), Sénèque *et al.* (2019) and Sauveroche *et al.* (2020). Sénèque *et al.* (2019) also recorded the posture of the horse *in situ.*

When behaviour in horses being kept in groups was recorded, the focus was on the type of social interactions observed and the extent to which individual behaviour was affected by other group members (feeding, drinking, and resting behaviours). Negative social interactions, primarily levels of aggression and avoidance behaviours were recorded in relation to different approaches to forage provision (Melvin *et al.* 2020; Sundmann *et al.* 2022; Baumgartner *et al.* 2023) and around water resources (Hildebrandt *et al.* 2022). Social disturbance of lying bouts was recorded by Burla *et al.* (2017) and by Hildebrandt *et al.* (2022). Both altruistic and different levels of agonistic behaviour were recorded by Suagee-Bedore *et al.* (2021) in relation to the size of the group turn-out area. The effect of one form of environmental enrichment (auto-brushes) on group-housed horses was assessed in terms of social interactions (both positive and negative) and facial expression when using the brushes by Lansade *et al.* (2022). See Table 2(b) for factors found to affect behaviour in the home environment.

#### Supporting evidence

The supporting evidence presented in the retained studies was variable. Reference to past studies and comparisons with physiological measures were most frequently used. Where horses were able to demonstrate choice or social interactions were recorded, approach behaviour and affiliative social interactions were deemed positive signs, while avoidance, and social disturbance/agonistic interactions were deemed negative signs (at least for the recipient). This attribution of affective state to different types of social interaction was supported by physiological measures in several studies. Suagee-Bedore *et al.* (2021) found a reduction in both plasma cortisol levels and agonistic/mild threat behaviours in relation to increased enclosure size. Sauveroche *et al.* (2020) found that increased positive social behaviour and resting behaviour both correlated with decreased concentrations of hair cortisol. Although Baumgartner *et al.* (2023) found both negative social interactions and salivary cortisol levels increased in the condition when there were fewer feeding stations, the authors noted that the level of physiological response was lower than in studies investigating the effect of other stressors (for example, transport). Similarly, Melvin *et al.* (2020) found that although agonistic threats and avoidance behaviour varied in relation to how forage was presented, no difference in plasma cortisol levels was found.

The effect of individual compared to group housing on behaviour and physiology was assessed in both adult horses and in youngsters stabled for the first time. Visser *et al.* (2008) recorded the response of young horses to first-time stabling, either individually or in pairs. Individually housed horses displayed more behaviours indicative of stress (neighing, pawing, nibbling, snorting), spent more time standing vigilant and sleeping, and less time eating than those in paired housing. At the end of the 12-week trial period, 67% of the horses housed individually were seen performing one or more types of stereotypical behaviour. The response to a corticotropin-releasing factor (CRF) challenge test was reduced in those stabled individually, suggested by the authors to be the result of a desensitisation of the hypothalamic-pituitary-adrenal (HPA) axis in response to the stress associated with isolation (Visser *et al.* 2008). Individually housed horses had higher mean heart rates in the stable than those in paired housing (Visser *et al.* 2008). Harewood and McGowan (2005) recorded more stress-related behaviours when fillies were individually housed for the first time than during the previous 24 h when in groups in an outdoor paddock but found no differences in salivary cortisol levels or heart rate. Foals housed in an enriched environment that included turn-out time in groups showed less vigilant/aberrant behaviour, and increased time lying and with ears forward than foals kept in a non-enriched stable environment with only individual turn-out (Lansade *et al.* 2014). After eleven weeks almost 50% of the foals in the control environment did not finish their feed. After six weeks higher levels of salivary cortisol were found in the morning in the enriched group (Lansade *et al.* 2014). These three studies highlight the complexity associated with using cortisol concentration as a means of interpreting behaviour. Young horses showed behavioural signs of negative affective state following a short-term housing experience, but no physiological response was recorded (Harewood & McGowan 2005). Longer-term exposure to a seemingly negative home environment was found to desensitise the HPA response by Visser *et al.* (2008) and Lansade *et al.* (2014) found increased cortisol levels associated with an enriched environment.

The effect of age on cortisol responses and the extent to which this physiological measure can be used to assess the valence of affective state as opposed to arousal is currently unclear. In adult horses, higher levels of faecal corticosterone were found when they were housed individually (as compared with paired or group housing), but the only behavioural differences recorded were an increase in feeding and a decrease in standing behaviour when horses were paddock-housed in a group, which could be accounted for by the availability of grass (albeit relatively bare) and space in this condition (Yarnell *et al.* 2015). Considerable individual differences were found when heart-rate variability (HRV) was monitored by Werhahn *et al.* (2012), but in general, this was lower in horses with no turn-out time, with these horses spending less time lying down in the stable than those who were allowed turn-out. The latter finding could be explained by an increase in feeding time and decreased resting time during turn-out.

As an additional means of assessing how the home environment related to affective state, additional tests were included in six of the retained studies. Visser *et al.* (2008) found no difference in response to a novel object test between the housing groups. Harewood and McGowan (2005) found that fillies were less relaxed during saliva sampling after being housed individually for 24 h (compared to when group-housed in a paddock). Yarnell *et al.* (2015) found that individually housed horses were less relaxed during temperature sampling than when group or pair housed. Less aggression towards humans was found by Ruet *et al.* (2019) when horses were housed in stables with a window to the outside and on straw (as opposed to non-straw) bedding. During ridden work, rider-assessed ‘willingness to perform’ was scored lowest in those horses with no turn-out (Werhahn *et al.* 2012). Similarly, when horses stabled in total confinement were assessed by riders after ridden police patrol work, they were deemed to be more alert, nervous, and fearful than when allowed partial turn-out (Pessoa *et al.* 2016).

#### Limiting factors

The retained articles relating to the home environment varied greatly in terms of objectives, experimental design and behaviour recorded. In addition, justification for attributing affective state to specific behaviours relied primarily on past work, with some findings being supported by means of physiological measures and/or responses to handling and ridden activities. However, when recording behaviour in different situations it is important to highlight the fact that certain behaviours can only occur if resources are provided to facilitate them (for example, social behaviour, movement, feeding behaviour) and these limitations must be accounted for. Also, in most studies there was a bias towards recording behavioural signs of negative affective state, with less frequent conclusions relating to behavioural signs of positive affective state.

Identifying behaviour *in situ* in the home environment that is indicative of positive affective state is limited by and dependent upon features of that environment. Where individual stabling, lack of space and limited access to forage are features of the home environment, the behavioural repertoire of the horse is severely restricted. Whether or not the subjective experience of horses kept in such an environment can ever be positive is debatable and different interpretations of similar behaviours were evident in this review. For example, ‘alert posture (elevated neck with ears pricked forward)’ was deemed a positive sign of interest in the environment by Ruet *et al.* (2019) and negative, vigilant behaviour by Lansade *et al.* (2014). A similar discrepancy was found in relation to whether increased time spent standing relaxed is a positive sign (Sauveroche *et al.* 2020), or increased time standing dozing a negative one (Visser *et al.* 2008) (see Table 2[a]). Such interpretations can both potentially be correct, as informed by the context, and the degree to which they occur, but this does highlight the challenge when attributing affective state to behavioural signs. An association between certain postural morphometric measurements taken when the horse was standing in the stable and whether the horse was reported as displaying stereotypical behaviour could inform differentiation between positive and negative signs (Sénèque *et al.* 2019), although potentially confounded by the type and conformation of individual horses. Behavioural signs of negative affective state were reported more consistently, and these included stereotypical behaviours (Visser *et al.* 2008; Ribeiro *et al.* 2019) and behavioural events such as ears backwards, sniffing, licking stable walls/bars, repetitive head swinging (Lansade *et al.* 2014), pawing, stamping, kicking at walls, and high-pitched whinnies (Visser *et al.* 2008; Lesimple *et al.* 2019), as shown in Table 2(a).

#### In summary

The results of the studies retained all support the conclusion that equine welfare is likely to be compromised by individual, restrictive housing. This was reflected in behaviour expressed *in situ* and in behavioural responses to handling and ridden tests (see also Part II; Hall & Kay 2024). The need for company and the negative consequences of lack of social contact were demonstrated in young horses where reduced feeding time and subsequently reduced weight gain were reported when they were housed individually as opposed to in pairs (Visser *et al.* 2008), and in adult horses in the study by Yarnell *et al.* (2015). Other features of the home environment found to be necessary for the horse to fulfil its behavioural needs, and by implication to impact on its affective state, included sufficient space and adequate forage provision. Specific factors reported in the retained studies are shown in Table 2(b).

### Intra-species social behaviour

A total of twenty articles that focused primarily on intra-species social behaviour were retained, including observational studies of established and/or free-ranging groups, and experimental manipulation of group membership and location, as well as those assessing features of social communication. Social interactions were used as a measure of group welfare and individual affective state in ten studies (Strand *et al.* 2002; Christensen *et al.* 2011; York & Schulte 2014; Górecka-Bruzda *et al.* 2016; Farmer *et al.* 2018; Majecka & Klawe 2018; Pierard *et al.* 2019; da Cruz *et al.* 2023; Kieson *et al.* 2023; Stachurska *et al.* 2023), with affiliative interactions being attributed with a positive valence and agonistic interactions a negative valence (at least in relation to the recipient). When compared with the studies reported in *The home environment* the sources cited for the development of ethograms by those reporting social behaviour were more consistent. Out of the fourteen sources cited, five studies referred to McDonnell and Haviland (1995), three to McDonnell (2003) and two referred to each of Waring (2008), Christensen *et al.* (2002) and Jørgensen *et al.* (2009). See Table S1.2 in the Supplementary material for further details of the retained studies. Intra-species social behaviour and communication indicative of affective state (positive or negative) and the supporting evidence is shown in Table 3(a).

**Table 3.** (a) Intra-species social behaviour and communication indicative of affective state (positive or negative), supporting evidence*, and (b) factors affecting this behaviour

	Signs of positive affective state	Basis of justification	Signs of negative affective state	Basis of justification
**3(a) SOCIAL BEHAVIOUR AND COMMUNICATION**				
**Behaviour of individual**				
Non–social activity			Increased time standing alert and autogrooming, increased activity, rolling, urination, defaecation. Decreased time spent grazing Increased locomotor activity (trotting, cantering, pawing)	–ve SIT (Strand *et al.* 2002) –ve SIT (Strand *et al.* 2002) –ve SIT (Górecka–Bruzda *et al.* 2022)
Behavioural events			Increased yawning	–ve SIT (Górecka–Bruzda *et al.* 2016)
**Intra–species interactions**				
Whole body	Fewer affiliative interactions (allogrooming) but of a longer duration Affiliative interactions (including allogrooming) occurred more frequently when recipients were positioned on the right side, where they lasted longer More affiliative interactions occurred when recipients were positioned on the left side	+ve SOC (Kieson *et al.* 2023) +ve SOC (da Cruz *et al.* 2023) +ve SOC (Farmer *et al.* 2018)	Increased frequency of affiliative interactions (allogrooming) but of a shorter duration Increased social play (adult horses) Non–contact agonistic behaviour (displacement, threat to kick, bite, chase, mouth clapping)	–ve SIT (Kieson *et al.* 2023) PIP, –ve SOC (Hausberger *et al.* 2012) –ve SOC (Christensen *et al.* 2011)
Facial expression	Positive attention (ears are up and rotated forward, eyes are directed forward, nostrils are moderately dilated, the mouth is usually closed)^a^	+ve SIT (Wathan *et al.* 2016)	Agonistic expression (muscles are tense, ears are rotated backwards and flattened to the skull, nostrils are usually dilated and drawn back/upwards, causing wrinkles along the upper posterior edge, lips are often pursed. In more extreme cases, mouth may be open, and incisors exposed in a bite threat)	–ve SOC (Wathan *et al.* 2016)
Vocalisations			Increased vocalisation	–ve SIT (Strand *et al.* 2002; Górecka–Bruzda *et al.* 2022)
	Differences in the frequencies, energy spectrum and duration of horse vocalisations have been shown to be associated with the valence and arousal level of the underlying emotion: +ve/–ve SIT (Pond *et al.* 2010; Briefer *et al.* 2015, 2017; Maigrot *et al.* 2017, 2022; Stomp *et al.* 2018a,b).			
**3(b) FACTORS AFFECTING SOCIAL BEHAVIOUR**				
Size of enclosure	Bouts of allogrooming with preferred partners occur less frequently and last for longer in open pasture settings, more frequent but shorter bouts in confined settings. Number of social interactions (both affiliative and agonistic) decrease as paddock size increases. Instances of contact and threat aggression positively linked to density of grouping			(Kieson *et al.* 2023) (Majecka & Klawe 2018) (Pierard *et al.* 2019)
Stability of group	Repeated re–grouping increased the occurrence of agonistic behaviour (mainly non–contact), play behaviour more variable in unstable group. Group changes increased agonistic behaviour in feral mares.			(Christensen *et al.* 2011) (Nuñez *et al.* 2014)
Feral vs domestic horses	Lateral preferences for affiliative behavioural interactions vary for feral/domestic horses – may relate to experience of human handling/training			(da Cruz *et al.* 2023)
Sex of horse	Mares were found to be more socially dependent than geldings, showing less motivation for food and more standing alert/activity when isolated.			(Górecka–Bruzda *et al.* 2022)
Attenuation of fear	A reduction in behavioural and physiological signs of fear in response to a startle test was found when naïve horses were in the presence of a habituated demonstrator.			(Rørvang & Christensen 2018)
Inter–species interactions	Relaxed open mouth observed during play behaviour between dogs and horses			(Maglieri *et al.* 2020)

* Key to supporting evidence: Past studies (PS), physiological measures (PHYS), assumption of associated pain (PIP), situations deemed positive or negative (+ve/-ve SIT), positive or negative social interactions (horse/human) (+ve/-ve SOC), approach/avoidance (APP/AVO), choice (PREF).

a *A neutral/relaxed facial expression is also described as follows*: Body and facial muscles are relaxed. Some eye closure. Ears may continue to move or, if very relaxed, will rest in a lateral position. Lower lip may droop (Wathan *et al.* 2016).

#### The physical environment

The impact of physical features of the home environment on social interactions was assessed by Majecka and Klawe (2018) and Pierard *et al.* (2019). An increase in both affiliative and agonistic interactions was associated with a decrease in enclosure size (Majecka & Klawe 2018), and aggressive threats increased with increases in stocking density (Pierard *et al.* 2019). Affiliative interactions (allogrooming) between preferred partners increased in frequency but were of a decreased duration in confined (n = 85) as opposed to open pasture (n = 115) settings in large established groups of mares (Kieson *et al.* 2023). Other behavioural signs found to be associated with negative valence (agonistic behaviour: Luz *et al.* 2015; discomfort: Torcivia & McDonnell 2021), including increased locomotion and vigilant stances were observed in the confined condition (Kieson *et al.* 2023). In addition to spatial differences in the studies by Majecka and Klawe (2018) and Kieson *et al.* (2023), the larger enclosures also provided more forage/grazing opportunities which are likely to have contributed to behavioural differences.

#### Group stability

Group instability and change resulted in an increase in agonistic interactions in young (two years of age) domestic mares (Christensen *et al.* 2011) and physiological signs of stress in feral mares that had been treated with porcine zona pellucida (PZP) contraception (Nuñez *et al.* 2014). In the latter study, the conclusion that these group changes had a negative impact on the mares was informed by an increase in faecal cortisol metabolites associated with these group changes (Nuñez *et al.* 2014). In domestic horses, an increase in standing alert and decrease in time spent grazing was found when mares were removed from their group and either isolated or kept with a single companion for a short period (6 h). However, no effect on plasma cortisol level or heart rate was found when compared with mares that had been returned to their group rather than put into isolation (Strand *et al.* 2002). Although increases in whinnying, urination and rolling were recorded, there was no difference between mares kept alone or with a companion, and the authors note that there were three factors that could account for these behaviours: the novelty of the environment, isolation, and separation from the established herd (Strand *et al.* 2002). Whether this short-term separation from the established group resulted in a change in social interactions when the mares rejoined the group was not reported.

#### Group structure and composition

In feral horses, affiliative interactions (including grooming) were associated with a right-side preference which the authors (da Cruz *et al.* 2023) suggested was linked to the left-brain hemisphere dominance for positive affect and affiliative behaviours (Ahern & Schwartz 1979). This finding contrasts with that of Farmer *et al.* (2018) where domestic horses were shown to have a left-side preference during allogrooming, a consequence perhaps of human handling which tends to occur more frequently from the left side of the horse. Differences between Przewalski (*Equus ferus przewalskii*) and domestic horses were found by Górecka-Bruzda *et al.* (2016) in relation to the association between social interactions and yawning behaviour. There was a positive correlation between the frequency of yawning and aggressive interactions in Przewalski horses and between yawning frequency and affiliative interactions in domestic horses. It was suggested that yawning could relate to the intensity, rather than the valence, of a social interaction (Górecka-Bruzda *et al.* 2016).

As well as differences between feral and domestic horses, other characteristics have been shown to affect the type and frequency of social interactions. Groups comprising ponies (Shetland and Welsh) had more friendly interactions than mixed horse groups (Majecka & Klawe 2018). Mares in oestrus were recorded as standing for longer and spending less time in locomotion than those in dioestrus, and an increase in the number of agonistic interactions occurred at the start of dioestrus (Stachurska *et al.* 2023). Less time was spent active, more time feeding, and fewer social interactions were recorded in lactating compared with non-lactating mares (York & Schulte 2014). Górecka-Bruzda *et al.* (2022) found mares to be more socially dependent than castrated males. In a two-choice test between access to food or a companion, more mares than geldings chose the companion over food. Such choice tests provide valuable insights into factors that are important to the horses and are likely to promote positive affect.

Two studies included observations of play behaviour in young (two years of age: Christensen *et al.* 2011) and adult (Hausberger *et al.* 2012) horses. Play behaviour varied between different groups of young horses and was even more variable in unstable as opposed to stable groups (Christensen *et al.* 2011). In adult horses, those exhibiting play behaviour were found to be more likely to also have potentially painful vertebral issues, and showed more aggression towards humans, than non-players (Hausberger *et al.* 2012), but further evidence is required before wider conclusions can be drawn in relation to underlying affective state.

#### Communication of affective state

The communication of affective state by means of vocalisation was reported in six retained studies (Pond *et al.* 2010; Briefer *et al.* 2015, 2017; Maigrot *et al.* 2017, 2022; Stomp *et al*. 2018b). Differences in vocalisation (whinnies) according to the valence of the situation were reported by Pond *et al.* (2010), and Briefer *et al.* (2015) and a specific vocalisation (snort) was reported by Stomp *et al.* (2018b) to be associated with positive experiences. Differences in the frequencies, energy spectrum and duration of horse vocalisations have been shown to be associated with the valence and arousal level of the underlying emotion, with physiological responses (HRV, RR) reliably changing with arousal level but not valence (Briefer *et al*. 2015). A weak (but inconsistent) association between both arousal and the valence conveyed within the vocalisation and changes in skin temperature (ST) was however found (Briefer *et al*. 2015). Species-specific differences may occur in the way that valence is communicated within vocalisations (Maigrot *et al.* 2017) but some cross-species recognition of both valence and arousal communicated through vocalisation has been demonstrated in the horse (Maigrot *et al.* 2022).

Valence has been associated with specific features of horse facial expression (Wathan *et al.* 2015), and when presented with 2D images of horse faces portraying positive or relaxed expressions, or agonistic expressions, chose to approach the former and avoid the latter (Wathan *et al.* 2016). Horses clearly use both auditory and visual information generated by conspecifics to guide social behaviour, facilitate positive interactions, and avoid aggressive encounters.

#### In summary

The physical and social environment combine to determine whether the species-specific needs of the horse can be fulfilled. There is a consensus that group membership is a priority for the horse and that the valence of social interactions contributes to and is evidence of individual affective state. Environmental conditions, group structure and composition, and individual characteristics have all been shown to relate to the type and frequency of social interactions occurring within that group, which in turn provide an insight into the affective state of group members. Behavioural signs of group cohesion and the nature of within-group social interactions reflect the quality of life of horses within a social group. Although short-term separation from the group was not found to have a negative impact on some individuals, it is not clear how such removals affect the overall stability of the group. In addition, behaviours associated with separation anxiety can cause safety issues for human handlers as well as being signs of distress in the horse (see Hartmann *et al.* 2011). Regardless, providing the horse with companionship in the form of a stable group of conspecifics is imperative to satisfy social behavioural needs. The potentially negative effect of removal from the group may be lessened by repeated short-term experiences, followed consistently by return to the group. Further evidence is required to determine how the negative impact of separation from the group can be reduced, but keeping horses alone is not an acceptable solution. Factors found to affect behavioural signs of affective state in social situations are shown in Table 3(b).

### Weaning

Fourteen articles relating to the behaviour of mares and foals during and after the weaning process were retained. The age at which foals were weaned varied from four and a half months (Henry *et al.* 2012) to approximately eight months (Lansade *et al.* 2018), with most weaning occurring at around six months of age. The process of artificial weaning in the domestic horse is acknowledged as being a negative experience for both mare and foal, and the focus of the retained articles was on how this experience could be managed to make it less stressful. The impact of periods of separation pre-weaning, abrupt or gradual weaning, and of different post-weaning groupings, were assessed using both behavioural and physiological measures. Time budgets and activity pre- and post-weaning were compared in both mares and foals, as well as social interactions and behaviour associated with stress (distress). Behaviour of the mare and foal following separation during weaning is shown in Table 4(a). Further details of the retained studies are included in Table S1.3 in the Supplementary material.

**Table 4.** (a) Mare and foal behaviour following physical separation at weaning, and (b) factors affecting this behaviour. The process of weaning is assumed to be a negative/stressful experience for both mare and foal. Behavioural responses of mare and foal observed immediately post-weaning and factors found to affect this behaviour are shown below. Where physiological measures have been reported this is indicated by PHYS

	BEHAVIOURS RECORDED POST PHYSICAL SEPARATION	SOURCE OF BEHAVIOURAL INFORMATION
**4(a) Behaviour of mare and foal**		
FOAL	Increased duration walking, pawing Increased running/activity Decreased time spent lying down Increased time spent standing, resting, lying (decreased activity) Decreased time spent feeding Increased vocalisation Increased frequency of defaecation Increased aggression towards other foals	(Moons *et al.* 2005) PHYS (Erber *et al.* 2012; Henry *et al.* 2012; Merkies *et al.* 2016) (Moons *et al.* 2005) PHYS (Wulf *et al.* 2018) PHYS (Delank *et al.* 2023) PHYS (Górecka–Bruzda *et al.* 2015; Wulf *et al.* 2018) (Moons *et al.* 2005) PHYS (Erber 2012; Merkies *et al.* 2016)) (Moons *et al.* 2005) (Merkies *et al.* 2016)
MARE	Increased locomotion, stall pacing, pawing Standing alert Decreased feeding Increased vocalisation Decreased aggression towards other mares	PHYS (Falomo *et al.* 2020) PHYS (Lansade *et al.* 2018) PHYS (Falomo *et al.* 2020) PHYS (Merkies *et al.* 2016; Lansade *et al.* 2018; Falomo *et al.* 2020) (Merkies *et al.* 2016)
**4(b) FACTORS AFFECTING BEHAVIOUR**		
Weaning process (abrupt / gradual)	Progressively weaned foals trotted and neighed less than abruptly weaned foals (and were more curious, gregarious, and less fearful for three months post–weaning). Mares rested more, were less active, stood alert less and neighed less following progressive weaning.	PHYS (Lansade *et al.* 2018)
Housing type during and after weaning	Weaning by confinement in a stable or barn increased the development of abnormal behaviours (stereotypic/redirected behaviours); housing in barns rather than at grass after weaning further increased the likelihood of abnormal behaviour developing.	(Waters *et al.* 2002)
Post–weaning group structure	Fewer behavioural signs of stress in foals housed alone at weaning compared with those housed in pairs. Foals weaned in groups that included two familiar, but unrelated adult mares showed less behavioural signs of stress. Foals weaned in groups including unrelated familiar adult horses vocalised less, did not behave aggressively to peers, or develop abnormal behaviours (such as excessive wood chewing and attempting to suckle on peers) when compared with foals weaned in a group that did not include adult horses.	PHYS (Hoffman *et al.* 1995) PHYS (Erber *et al.* 2012) PHYS (Henry *et al.* 2012)
Sex of foal	Fillies were more active and lay down less than colts Colts vocalised and defaecated more frequently than fillies More weight loss in fillies than colts over the first five days post–weaning and higher salivary cortisol concentration.	(Moons *et al.* 2005) PHYS (Wulf *et al.* 2018) PHYS (Wulf *et al.* 2018)
Familiarity with domestic housing	Immediately post–weaning foals that had been forest reared showed less locomotion, alert behaviour, and social activity, but stood still for longer than stable reared foals.	PHYS (Górecka–Bruzda *et al.* 2015)
Individual variation	Individual variation in behavioural response to separation in both mares and foals Stable individual variation in response to separation from mares shown by pre–weaned foals (from first postnatal week to six months)	PHYS (Merkies *et al.* 2016) (Pérez Manrique *et al.* 2019)
Diet	Foals fed a fat and fibre diet from one month cantered less frequently and for a shorter duration immediately post–weaning; and approximately two months post–weaning investigated a novel object for longer and were less averse to human contact/handling than foals fed a starch and sugar diet. Feeding concentrates after weaning increased the development of crib–biting.	(Nicol *et al.* 2005) (Waters *et al.* 2002)
Time since weaning	The behaviour of foals was not found to have returned to the pre–weaning profile three weeks post–weaning.	PHYS (Delank *et al.* 2023)
Provision of surrogate foal image	Some mares showed less behavioural signs of anxiety when separated from their foal (pre–weaning) when in the presence of a free–standing plywood image of a foal.	(Rogers *et al.* 2012)

#### Pre-weaning mare-foal separation

The effect of short-term (10-min) physical but not auditory separation of mare and foal at two, four, six, eight, ten and twelve weeks of age, on maternal care and subsequent response to weaning was assessed by Moons *et al.* (2005). Post short-term separation both mare and foal looked at each other more frequently and the foal initiated physical contact with the mare more frequently than pre-separation. At weaning there was no difference in HR response between foals that had been separated (n = 5) and the control group (n = 5), but the latter showed more locomotion. The foals that had been exposed to short-term separation had higher concentrations of salivary cortisol on the post-weaning morning (Moons *et al.* 2005), possibly a response to the failure to be reunited after this separation. Consistent individual variation in the response of foals to pre-weaning separation from the mare (for 2 min) as tested four times from one week post-natal to six months was found by Pérez Manrique *et al.* (2019) in both behaviour (vocalisation rate, raised tail and locomotion) and physiology (HRV). Due to the small sample size and contradictory behavioural and physiological findings by Moons *et al.* (2005) and the individual variation in foal response to maternal separation, with no subsequent measures taken following weaning (Pérez Manrique *et al.* 2019), there is no conclusive evidence that pre-weaning separation reduces the stress of the final physical separation.

A two-stage approach to weaning was trialled by Merkies *et al.* (2016) with nutritional separation (udder cover fitted to the mare to prevent nursing) for four days prior to physical separation. During the period of nutritional separation, foals were found to spend more time lying down than controls, but no behavioural or physiological differences between groups were found after physical separation. The only difference found between the groups was in the mares, where nutritional separation resulted in less vocalisation following physical separation. After physical separation, all foals were recorded as vocalising more, were more active and aggressive, and spent less time lying down. Increases in faecal cortisol metabolites were recorded in all mares and foals following physical separation, but individual variation in both behavioural and physiological responses was again found (Merkies *et al.* 2016). A gradual increase in the time spent physically separated (by a fence that allowed mares and foals visual, olfactory, and tactile contact, but prevented suckling) over the month preceding weaning was shown to reduce time spent trotting and vocalising compared with foals weaned without this preparation, as well as resulting in reduced concentrations of salivary cortisol (Lansade *et al.* 2018). This gradual weaning process also benefited the mares who showed fewer behavioural signs of stress and lower salivary cortisol levels than mares whose foals had been weaned without this progressive separation (Lansade *et al.* 2018).

#### Physical and social environment

The physical and social environment has been shown to be one key factor in how foals cope with artificial weaning and in their long-term well-being. For example, the development of stereotypical behaviour has been associated with confinement and isolation of foals during the weaning process (Waters *et al.* 2002). Regardless of how weaning is managed, changes in foal behaviour include increased vocalisation, locomotion, and reduced feeding, but these can be less pronounced if foals remain in a familiar environment and familiar group with known adult (unrelated) mares, compared with foals kept in peer groups without an adult (Erber *et al.* 2012). Foals moved to an unfamiliar environment at weaning, with some unfamiliar group members (foals) were found to spend an increased time lying down initially, followed by resting standing for longer periods than before they were weaned (Delank *et al.* 2023). A similar impact of an unfamiliar stable environment on general apathy in foals at weaning was found by Górecka-Bruzda *et al.* (2015), with an increased time spent standing, decreased social activity and reduced feeding recorded in forest-reared compared with stable-reared foals.

In contrast with the findings of Visser *et al.* (2008) (see *The home environment*), Hoffman *et al.* (1995) recorded fewer behavioural and physiological signs of stress in foals housed individually at weaning than in those housed in pairs. Foals housed in pairs stood still less than those housed individually (deemed a negative sign by Delank *et al.* 2023), with a high frequency of aggressive interactions recorded (Hoffman *et al.* 1995). The increased cortisol response in pair-housed foals may have related to the increased arousal associated with social interactions, regardless of valence. Lack of social contact during periods of isolation was found to reduce outward behavioural signs of emotional reactivity in yearlings (Lansade *et al.* 2012, see Part II; Hall & Kay 2024). The association between having a companion (or not) and the extent to which emotion is communicated behaviourally may explain in part the findings of Hoffman *et al.* (1995). Also, Henry *et al.* (2012) found that foals kept in groups that included unrelated (and in this case, unfamiliar) adult horses, rather than in peer-only groups vocalised less and had lower salivary cortisol responses. Only foals kept in groups without an adult horse showed increased aggression towards peers and abnormal behaviour such as wood-chewing (Henry *et al.* 2012).

#### Sex-related differences

Although there has been shown to be individual variation in the responses of mares and foals, artificial weaning is always associated with both behavioural and physiological signs of stress. Some differences between fillies and colts in both behaviour and physiological responses were found. Colts spent more time eating than fillies (Hoffman *et al.* 1995), and on the day of weaning walked less and defaecated more than fillies (Moons *et al.* 2005; Górecka-Bruzda *et al.* 2015; Wulf *et al.* 2018). Subsequently, colts demonstrated more curiosity about the environment, were more socially interactive and lay down less often than fillies (Górecka-Bruzda *et al.* 2015). Although initial weight loss was found to be greater in fillies than colts by Wulf *et al.* (2018), contrary to other studies, they did not find any sex-related differences in feeding or lying behaviour. However, levels of salivary cortisol (Wulf *et al.* 2018) and faecal cortisol metabolites (Górecka-Bruzda *et al.* 2015) were both higher in filly than colt foals at weaning. Despite many factors varying between studies, and some findings being based on relatively small sample sizes, there is evidence that there are sex-related differences in how foals respond to artificial weaning.

#### In summary

The behavioural and physiological response to artificial weaning cannot be avoided but the process can be made less onerous by adopting protocols that have been shown in the retained studies to lessen the negative impact of physical separation. The gradual approach to mare and foal separation outlined by Lansade *et al.* (2018) had much to recommend it. A priority should be to ensure that the weanling foals are kept in social groups that are familiar to them, including familiar adults, and in an environment that is also familiar to them. Although potentially challenging to many horse breeders, the short- and long-term well-being of these youngsters must be the primary concern. In agreement with Górecka-Bruzda *et al.* (2015), feeding behaviour is an indication of how foals are coping with separation from the mare, and potentially how mares themselves are coping. It is likely that mares will not be as adversely affected by environmental changes, but the provision of a stable social group and the freedom to move and feed are important factors that are likely to reduce the negative impact of separation from their foals. Factors affecting behaviour associated with weaning are shown in Table 4(b).

### Experimental tests of affective state

One approach used to objectively assess affective state in animals is to test whether they make a positive or negative decision relating to an ambiguous stimulus, termed a judgement bias test. Such tests have been used to assess the impact of housing and management (and consequently the extent to which species-specific needs were fulfilled) on underlying affective state and were subsequently included in Part I of this review. A total of eight such studies were retained, all of which reported the findings of spatial judgement bias tests (where the animals were trained to associate the location of the stimulus, usually a bucket, with a food reward or no food reward, and then presented with a stimulus in an ambiguous position). The results of these studies were inconsistent.

Two of the studies investigated the impact of current housing and management on whether horses demonstrated positive or negative expectations when presented with a stimulus in an ambiguous position (optimistic or pessimistic behaviour). Henry *et al.* (2017) found that those horses kept in a free-ranging social group displayed a positive judgement bias, whereas those kept in a more restrictive system in a riding school displayed a negative judgement bias. Although Marliani *et al*. (2022a) found some evidence of a more positive judgement bias in horses kept in an ethological stable system (Big Box©) compared to those in traditional stabling or in a more ‘natural’ open system, conflicting results from other measures (faecal and hair cortisol, personality traits, body condition scores) and confounding factors meant that no overall conclusions could be drawn. A similar lack of correlation between physiological measures and the results of judgement bias testing was found by Marliani *et al.* (2022b), although they identified individual differences associated with personality traits on the latency to approach certain stimulus positions. However, different personality tests were used in these two studies.

Two studies assessed the impact of management change on affective state as demonstrated by performance in judgement bias tests. Löckener *et al.* (2016) found a positive judgement bias in horses previously kept in individual housing with no access to pasture (for six months) after they had been moved to pasture and group living (for at least ten days). McGuire *et al.* (2018) found that horses (and one donkey) that had been rescued from abuse/neglect approached ambiguous stimuli more rapidly than those that had not experienced abuse/neglect and consequently had not been rescued.

#### Limitations of this approach

Although judgement bias testing offers a degree of insight into the affective state of animals, it requires prior training (often a lengthy process), and performance has been found to be affected by previous approaches to training. The effect of training using positive or negative reinforcement on judgement bias was assessed by Briefer-Freymond *et al.* (2014), with the finding that those previously trained using negative reinforcement made more positive appraisals of ambiguous stimuli than those trained using positive reinforcement. The food reward used in the judgement bias testing may have constituted a change for the better for those that had previously been negatively reinforced (Briefer-Freymond *et al*. 2014).

The search for more immediate objective measures of affective state has included comparisons with performance in judgement bias tests. A study evaluating eye wrinkles in horses during a judgement bias test found no significant association between these and evidence of optimism/pessimism based on performance (Hintze & Schanz 2021). However, Marr *et al.* (2018) found a significant relationship between motor laterality and performance in judgement bias tests, with a positive judgement bias being displayed by horses that moved off using the right forelimb first. In the study by Marliani *et al.* (2022b), horses that had been trained that the rewarded stimulus position was on the right were found to approach the ambiguous stimulus faster than those horses trained to reward from the stimulus position on the left. In terms of more general applicability, such approach behaviour in non-experimental situations could be used as an indication of positive expectations, potentially linked to a positive mental state. However, whether lateral biases or facial expression can provide any further insight is yet to be determined.

#### In summary

The results of the retained studies involving judgement bias testing provide some further evidence that housing and management that fulfils the need for space and company is associated with a more optimistic (positive) appraisal of ambiguous stimuli by the horse. Although there were confounding factors identified, and a lack of correlation with other measures, studies did demonstrate that change for the better (in terms of addressing the behavioural needs of the horse) was associated with optimism and potentially positive affective state. See Table S1.4 in the Supplementary material for further details of the studies retained.

### Stereotypical behaviour

Stereotypical behaviours are defined as being repeated, relatively invariant sequences of movement with no obvious function (Broom & Kennedy 1993), and not observed in free-ranging feral horses (Dodman *et al.* 2005). Management factors most associated with the development of stereotypies in stabled horses include housing conditions, feeding practices, and weaning method (Sarrafchi & Blokhuis 2013). In addition to the housing-related development of repetitive/stereotypical behaviour in young horses reported in *The home environment* (Visser *et al.* 2008; Lansade *et al.* 2014), seven articles were retained that reported factors associated with stereotypical and other unwanted stable-based behaviours.

#### Factors relating to the occurrence of stereotypical behaviour

Arena *et al.* (2021) compared the current housing and management of horses reported by a veterinarian as displaying behavioural pathologies (oral stereotypies, locomotor stereotypies, social problems, and training behaviours), with those not displaying such behaviour (and deemed healthy). A positive correlation between the occurrence of behavioural pathologies, time spent in the stable and frequency of work, and a low-fibre, high-energy diet was found. Justification for deeming such behavioural pathologies to be signs of chronic stress and potentially negative affective state was based both on the findings of previous studies and on higher plasma cortisol levels compared with healthy horses. No difference in hair cortisol levels between horses displaying behavioural pathologies and their healthy counterparts was found (Arena *et al.* 2021).

It has been suggested that the type of work/role of the horse affects the prevalence and type of behaviour exhibited. Zuluaga *et al.* (2018) found behavioural signs of chronic stress (including wood chewing, pawing, bed eating, crib-biting, and windsucking) in urban police patrol horses in Columbia, but it was not clear whether this was a direct consequence of the role or management factors. Although differences between horses involved in different sporting disciplines (including dressage, showjumping, eventing, and endurance) have been found (in some cases independently of management differences, for example, Hausberger *et al.* 2009), it is not clear whether these differences result from initial selection for a role or because of the demands of that role. Hanis *et al.* (2021) found differences between horse working groups (leisure, endurance, patrol horses) in the prevalence of abnormal/stereotypic behaviour in the stable, but also related differences in dietary intake between groups, and concluded that this was a major factor in the development of such behaviour. In general, the effect of work/role on behaviour is confounded by many factors (including selection, training, and management) and findings should consequently be carefully evaluated in terms of all contributory variables. However, as confirmed by the findings of a recent survey by Kádár *et al.* (2023), the prime factor is the type of home environment, with an increased occurrence of abnormal repetitive behaviours reported in stabled horses as compared with those kept at pasture.

#### The association between stereotypies and other features of behaviour

The impact of performing stereotypical behaviours on learning performance has been evaluated. No effect of stereotypical behaviour on learning ability has been found (Briefer Freymond *et al.* 2019, 2020), with the proviso that the performance of the stereotypical behaviour is not restricted. Horses that displayed crib-biting during a spatial learning task showed fewer behavioural signs of frustration and had lower levels of salivary cortisol than stereotypical horses that did not display this behaviour (Briefer Freymond *et al.* 2020). An increased sensitivity to tactile stimuli was found in crib-biting horses, which the authors suggested may be linked to physiological responses to chronic stress, that also potentiated the development of this behaviour (Freymond *et al.* 2019).

#### In summary

Regardless of the potential positive or negative impact that performing abnormal repetitive behaviours has on an individual’s QOL, research to date provides conclusive evidence that it is the failure to provide for the behavioural needs of the horse that is the causative factor. Although it is not clear whether the performance of stereotypies *per se* is a sign of current QOL, or a coping response resulting from past sub-optimal conditions (Sarrafchi & Blokhuis 2013), such behaviour has been shown to develop in young horses exposed to inappropriate housing and/or diet and persists throughout adulthood. As such, this behaviour provides evidence of the importance of providing for the behavioural needs of the horse throughout their life. Despite this, many horses continue to be managed in a way that fails to do so. See Table S1.5 in the Supplementary material for further details of the studies.

### Behavioural evidence of affective state

Despite the incorporation of positive welfare states in the extended Five Domains model used to assess animal welfare (Mellor & Beausoleil 2015), and the relative increase in articles identified by Search 1 (emotion, affect, cognition) compared with Search 2 (stress) post-2015, the focus of most studies was on behavioural signs of negative affective state, with an absence of negatively attributed behaviours being considered as a positive sign. Behavioural expression is inextricably linked to the physical and social environment, with most approaches to horse management providing little opportunity for the horse to display behaviours indicative of positive affective state, at least *in situ.* Inferences regarding behavioural signs of affective state in the home environment (see Table 2[a]) can be supported with reference to the two components of arousal and valence identified by Mendl *et al.* (2010). Behaviours considered to reflect positive affective state included standing calmly, recumbency (low arousal, positive valence: relaxed) and increased time spent feeding, as well as affiliative social interactions with conspecifics and allogrooming (high arousal, positive valence: reward-seeking). Clearly, such positive behavioural signs are dependent upon resource provision and social opportunities. Behaviours considered to reflect negative affective state included pawing, stamping, and kicking at the wall, and other repetitive behaviours including stereotypies, agonistic and avoidance behaviours (high arousal, negative valence), and withdrawn, unresponsive behaviour (low arousal, negative valence).

Reliance on the findings of previous studies and the exclusive recording of behaviours deemed indicative of stress were in some cases how conclusions regarding affective state were arrived at. As noted above, the focus of most studies was on behavioural signs of negative affective state, with an absence of negatively attributed behaviours being considered as a positive sign. This conclusion is not necessarily warranted, and whereas active attempts at escaping from an unsatisfactory situation may be unwelcome for the human carer, they do provide a clear signal that whatever is causing this response is something that the horse is objecting to. A lack of such behaviour may not suggest that the situation is acceptable, rather that the horse has given up trying to escape. General unresponsiveness has been associated with poor condition, poor health, exhaustion, chronic pain, and depression in horses (Burn *et al.* 2010). When exposed to repeated, unavoidable, aversive experiences, where there is no association between behaviour and the outcome for the horse, there is evidence that a condition akin to learned helplessness can ensue (Hall *et al.* 2008).

In addition to reference to past studies, and/or ethograms listing behaviours typical of a specific (generally negative) affective state, physiological measures were frequently used as a means of providing objective evidence to support this interpretation. Although consistency between physiological measures of stress (including HR, HRV, rectal temperature and cortisol levels) and behaviour was found in scenarios that were intrinsically negative experiences (for example, weaning), this was not always the case in other situations. Physiological measures have been shown to relate more closely to arousal than to the valence of an emotional response (Hall *et al.* 2018) and other factors may be influencing the type of behavioural response elicited. For example, Squibb *et al.* (2018) attributed the lack of correlation between physiological and behavioural responses in adult horses during a novel task to different levels of training (see Part II: *Tests of emotional reactivity*; Hall & Kay 2024). Although the findings of individual studies have varied, and physiological responses have been associated with factors deemed to be negative experiences for the horse (for example, individual housing: Yarnell *et al.* 2015), the relationship between physiological response, behaviour, and affective state requires further investigation.

**Figure 3.**
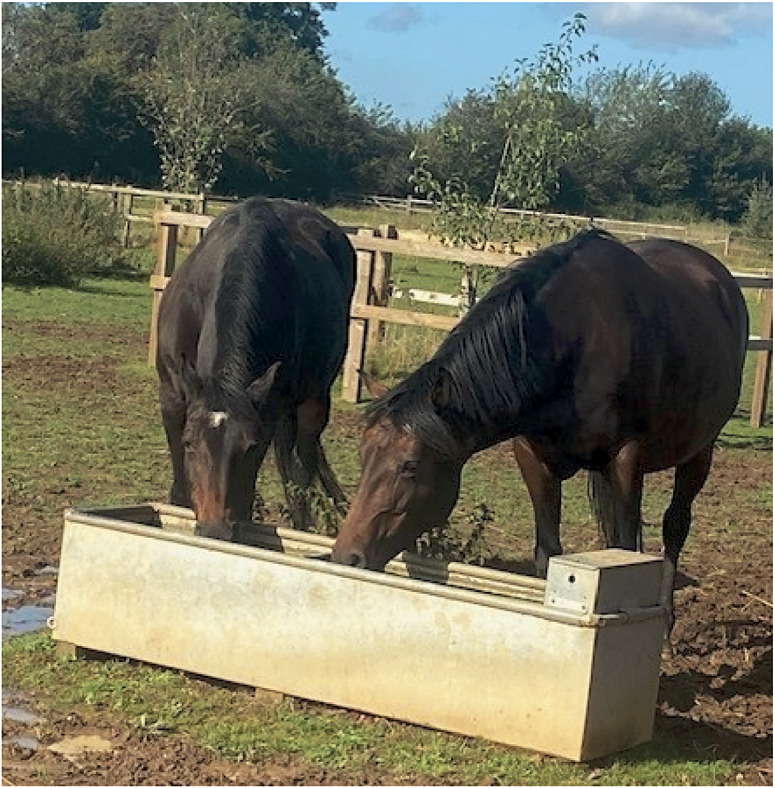
Amicable sharing of resources such as water is a positive sign of group cohesion and well-being in domestic horses.

In addition to behaviour *in situ*, features of the home environment and the provision of forage related to behaviour in other scenarios. For example, ease of handling was facilitated by group/paired housing (Harewood & McGowan 2005; Yarnell *et al.* 2015) and provision of turn-out resulted in an increase in willingness to perform (Werhahn *et al.* 2012) and a reduction in nervous/fearful behaviour (Pessoa *et al.* 2016) when ridden. Behavioural signs of affective state in groups of horses were predominantly assessed by comparing the occurrences of agonistic and affiliative interactions, with the latter considered a positive sign. The QOL of individual group members is likely to be affected by the cohesiveness of the group, amicable sharing of resources (for example, water, see Figure 3), and potentially the social facilitation of activity patterns, for example, group recumbency (K Griffin, personal communication 2023). Further evidence is required to determine the relationship between group behaviour and the QOL of individual group members.

### QOL in breeding stock

Although multiple articles relating to most aspects of the life of the domestic horse were identified by the search terms, there were a few notable omissions. Despite the potential for the management of breeding stock to negatively impact the QOL of both mares and stallions (and subsequently their offspring), no articles specifically relating to this scenario were identified by the searches. This lack of evidence within the scientific literature regarding the welfare of breeding horses was noted by Campbell (2017), alongside the need to align methods of husbandry more closely with the natural reproductive behaviour of the horse (Waran 1997; Campbell & Sandøe 2015). A review of the aspects of stallion management that contribute to their quality of life, or lack thereof, concluded that the way many stallions are currently housed and managed results in a lack of social skills, increased aggression, and stereotypic behaviour (de Oliveira & Aurich 2021). The home environment and social opportunities afforded to many stallions (Popescu *et al.* 2019), as well as the welfare problems associated with the way horses are bred (Campbell & Sandøe 2015) require urgent consideration to promote better quality of life for horses within the breeding sector. The findings of this review have highlighted the importance of social stability and access to an appropriate physical and social environment from birth onwards. In addition to the provision of friends, freedom, and forage (Fraser 2012), the importance of family to the horse should also be considered.

### Animal welfare implications

The results of Part I of this review provide extensive evidence that the first stage in moving towards a better quality of life for horses is to ensure that throughout their lives the home environment satisfies their need for company, freedom of movement and environmental choices, as well as access to adequate forage. Failure to provide for any of these basic species-specific needs will undermine attempts to achieve a good life for horses. Current findings wholly support those of the early study by Schatzmann (1998) where the highest priority of horses was always to be in the company of, or within view of, other horses and to be eating forage outside.

Although the horse’s need for space, company and forage has long been acknowledged, many are still stabled individually and have restricted access to all three. Current findings support the conclusions of Ruet *et al.* (2019) that an optimum environment should allow for free exercise, interactions with conspecifics and *ad libitum* fibre availability to facilitate positive affective state in horses and ponies. Opportunities for free exercise, as provided by paddock turn-out, were instrumental in increasing signs of relaxation in horses (Pessoa *et al.* 2016), reducing the occurrence of behavioural pathologies (Arena *et al.* 2021) and promoting increased interest in the environment (Ruet *et al.* 2019). The impact of having access to both space and company was demonstrated in the study by Löckener *et al.* (2016), where an optimistic state of mind was generated in horses tested in a judgement bias task once they had been moved from restrictive individual stabling to being kept in a group on pasture. Housing in company, ideally in a stable group, was shown to be associated with positive behavioural signs from an early age and throughout adulthood. Paired housing (as opposed to individual housing) in young horses stabled for the first time was less likely to result in behavioural signs of stress and the development of stereotypical behaviour patterns (Visser *et al.* 2008) and was associated with increased time spent eating forage in adult horses (Yarnell *et al.* 2015).

The social group in which horses live plays a major part in determining their general welfare. Comparisons with feral horse groups are a valuable approach to further understand what constitutes an appropriate group structure (Waran 1997), although in most cases domestic constraints will limit the extent to which this can be applied. Group structure has been found to be of particular importance in foals, where the presence of unrelated adult horses was found to reduce the stress associated with weaning (Erber 2012; Henry *et al.* 2012). This reflects the social structure in feral horses and the presence of older mares would be likely to exert a calming effect on foals via emotional transfer, as well as contributing to the social learning of foals that will prepare them for future group living. Also, feral group structure may explain why mares (Górecka-Bruzda *et al.* 2022) and fillies (Moons *et al.* 2005; Wulf *et al.* 2018) were found to show a greater level of distress than their male counterparts in response to separation and/or isolation. The survival of feral mares and their offspring is dependent upon the stability of the family group (harem), whereas male groups are much more transitory (Waran 1997). Optimum grouping is a necessity for the development of foals and young horses, and the relative impact of change on individual horses should be considered when management decisions are made.

When applying the Five Domains model (Mellor & Beausoleil 2015) to assess the adverse impacts of husbandry and training on horse welfare, nutrition was considered by a panel of experts to have the least bearing on resultant subjective mental state when compared with other behavioural restrictions (McGreevy *et al.* 2018). However, the findings of this review suggest otherwise. The provision of adequate forage has been demonstrated as integral to promoting horse health and positive affective state (Ruet *et al.* 2019). In stabled horses, long gaps between meals and inadequate forage were associated with an increase in the development of unwanted behaviours, including stereotypies (Ribeiro *et al.* 2019). Providing forage in a way that allows sufficient space for all individuals to access forage with minimal conflict in a group housing situation reduced the occurrence of agonistic behaviour and increased group cohesion (Sundmann *et al.* 2022; Baumgartner *et al.* 2023). Feeding is classed as a behaviour that has critical short-term consequences for an animal, is maintained for as long as possible and is highly resilient to change (Hall *et al.* 2018). As such, the need to consume sufficient forage and for an adequate time is a prime motivating factor for the horse. Indeed, as reported by Bulens *et al.* (2015) (see also Part II; Hall & Kay 2024), when forage was available horses were less inclined to engage in other behaviours with lower resilience, such as exploring the environment and investigating novel objects. The importance of fibre in the diet of foals from one month was evident from the comparative effect of a fat and fibre diet or a starch and sugar diet, with the latter being associated with more behavioural signs of distress post-weaning and increased aversion to human handling (Nicol *et al.* 2005). A low-forage diet was also associated with health issues and potentially a source of abdominal pain, as discussed in Part II of this review (Destrez *et al.* 2015; Hall & Kay 2024).

There were a number of general limitations associated with this review which are discussed in Part II (Hall & Kay 2024), but these do not detract from the unavoidable conclusion that to ensure a good QOL for all horses, the equine sector must urgently review the way horses are managed and implement change to ensure that their species-specific needs are met. Too long have human requirements been prioritised over the needs of the horse, but this must now be addressed to ensure that the horse-human partnership is mutually beneficial.

## Conclusion

The potential for horses to live a good life requires them to be provided with opportunities to fulfil their species-specific needs. Current approaches to horse management frequently fail in this respect and consequently, their quality of life is sub-optimal. Behaviours indicative of negative affective state, often resulting from inadequate social and physical environments, in addition to insufficient forage provision, were evident both in the home environment and in other scenarios, including during interactions with humans. Such behavioural signs included withdrawn or agitated behaviour *in situ*, avoidance behaviour during handling procedures, and agonistic intra-specific social interactions. Although the lack of such behavioural expression may be considered by many in the equine sector as evidence that the horse has an acceptable QOL, the findings of this review suggest otherwise. The focus of many of the studies was to assess the relative inadequacies of approaches to management and the impact of these on the behaviour of the horse. Although fewer negative behavioural signs may be associated with a reduction in the extent to which the horse experiences a situation as unpleasant, positive behavioural signs of pleasure were less frequently identified. The potential for behaviour indicative of pleasure to occur was dependent upon resource provision. Affiliative intra-specific social interactions, approach behaviour indicative of positive anticipation of a reward, and forage ingestion were all considered positive signs. Although a calm demeanour *in situ* was interpreted as positive, this may just be a sign of acceptance rather than pleasure.

The first stage in moving towards a better life for horses is undoubtedly to provide them with the opportunity to satisfy their behavioural needs from birth onwards and improve their potential for experiencing pleasure. The findings of the first part of this review provide evidence of what horses want and indeed need to live a good life, and how behaviour reflects their QOL. The impact of the many and varied interactions with humans on horse QOL is discussed in Part II (Hall & Kay 2024).

## Supporting information

Hall and Kay supplementary material Hall and Kay supplementary material

## Supplementary material

The supplementary material for this article can be found at http://doi.org/10.1017/awf.2024.38.
