# Supplementary material for: Living the good life? A systematic review of behavioural signs of affective state in the domestic horse (Equus caballus) and factors relating to quality of life. Part I: Fulfilment of species-specific needs
Source: Anim Welf. 2024 Oct 21;33:e40. doi: 10.1017/awf.2024.38 (PMC11503716; doi:10.1017/awf.2024.38)
Supplement: Hall and Kay supplementary material [file S0962728624000381sup001.docx]

**Supplementary Material (Part I)**:

Contents

1. Key to Tables S1.1-S1.5 (study design features)
2. Tables S1.1-S1.5 (with related reference lists of retained studies as referred to in each Table)

**1. Key to Tables S1.1-S1.5 (study design features)**

Sex of subjects: Mare (M), gelding (G), stallion (S), colt (C), filly (F)

Study design: Repeated measures (RM)

Between subjects (BS)

Testing associations (TA)

Observational (OB)

Type of ethogram used to record behaviour: E1 Specific behaviour recorded Specific behavioural characteristic recorded and general ‘emotion- related’ ethogram not relevant (sound quality / vocalisation details, laterality, performance / response to specific task, food sampling behaviour, personality assessment)

E2 List of behaviours (no description) a. List of behaviour with no reference to previous work

b. List of behaviour with reference to own previous work

c. List of behaviour with reference to other previous studies

E3. Behaviour and descriptions a. Full ethogram with no reference to previous work

b. Full ethogram with reference to own previous work

c. Full ethogram with reference to other previous studies

Supporting evidence presented to justify conclusions re: behaviour: Reference to past studies (PS)

Physiological measures (PHYS)

Pain inducing procedures / evidence of pain (PIP)

Situation regarded as positive (+veSIT) or negative (-veSIT)

Positive (+veSOC) or negative (-veSOC) social interactions

Approach (APP) or avoidance (AVO) behaviour

Preferences tested / choice (PREF)

Specific physiological measures*: Heart rate (HR), heart rate variability (HRV)

Blood pressure (BP)

Plasma cortisol (PC), salivary cortisol (SC), faecal cortisol metabolites (FC), hair cortisol (HC)

Eye temperature (ET), Ear-pinna temperature (EPT)

Rectal temperature (RT)

Skin surface temperature (ST)

Respiration rate (RR)

Salivary oxytocin (SOX)

* Other physiological measures specific to individual studies are not included in the tables below.

NA signifies not applicable.

**2. Tables S1.1-S1.5 (with reference lists of retained studies as referred to in each Table)**

- S1.1 Behaviour in the home environment
- S1.2 Intra-species social behaviour
- S1.3 Mare and foal behaviour during weaning
- S1.4 Experimental tests of affective state (judgement bias testing)
- S1.5 Stereotypical behaviour

**Table S1.1: Study details for the 21 retained articles relating to behaviour in the home environment**

| **Study** | **Focus of study &**  **other tests included** | **Study design** | **Length of study** | **Subject details** | | | **Behaviour recorded** | **Type of ethogram**  **used** | **Supporting evidence** | **Physiological measures** |
| --- | --- | --- | --- | --- | --- | --- | --- | --- | --- | --- |
|  |  |  |  | **No.** | **Age** | **Sex** |  |  |  |  |
| Baumgartner et al. 2023 | Number of feed stations | RM | 3 weeks (4 conditions each observed twice) | 28 | 6-23yrs | M,G | Aggression / risk of injury when feeding in groups | E3c | -veSOC, PHYS | SC |
| Burla et al. 2017 | Bedding area, stable size | RM | 4x11 days, recorded last 3 days of each treatment | 38 | 1–22yrs | M,G | Frequency / duration lying bouts / social disturbance | E3c | -veSOC |  |
| Greening et al. 2021 | Bedding depth, spatial memory | RM | 5 weeks, 6 days per treatment | 10 | Average 14.9yrs | M,G | Sleep states | E3b | PS |  |
| Harewood & McGowan 2005 | Out/indoor, group/individual, saliva sampling | RM | 24 hours in each housing type | 6 | 2yrs | F | Stress related behaviour in housing  Responses to saliva sampling | E3a  E3a | PS  AVO | SC |
| Hildebrandt et al. 2022 | Group use of resources | OB | 9 months: Water use 2 consecutive days/month; lying 18 days in 2 months | 51 | 2-29yrs | M,G | Social interactions at water  Resting behaviour | E3c  E3c | -veSOC |  |
| Lansade et al. 2022 | Environmental enrichment (EE) (auto-brushes) | OB | 6 weeks – behaviour recorded last 2 weeks | 40 | Average 7.5yrs | M | Social interactions  Facial expression when using EE | E3a  E3b | +veSOC  PS |  |
| Lansade et al. 2014 | Enriched stable environment | BS | 5 weeks in EE; then learning and personality tests (6-11 & 23 wks) | 19 | 10 months | F,C | Selected behaviour in EE compared with controls | E2a | APP  PS  PHYS | SC |
| Lesimple et al. 2019 | 2 complementary studies (stall architecture) | RM / TA | Long term effect of stabling >6 mnths | 32/23 | 6-19yrs | G/M,G | Stereotypical excitation and resting behaviour in relation to housing | E3c | PS |  |
| Marliani et al. 2021 | Stable type | OB | Sampled over 6 mnths | 5 | 11-16yrs | M,G | Activity budgets | E3c | PS |  |
| Melvin et al. 2020 | Forage presentation | RM | 7 wks - 2wks per condition Recorded wk 2 / 4. Pref. wk 6 or7. | 10 | Adult | M,G | Agonistic threats, aggression, avoidance and feeding behaviour at feed stations | E3a | -veSOC, PREF, APP, AVO | PC |
| Pessoa et al. 2016 | Effect of turnout, police patrol | RM | 4 mnths, 16 days per condition x2 | 8 | Average 9.5yrs | M,G | Behaviour in home environment and during ridden mounted patrols | E3c  E3c | PHYS, +veSOC, PS | HR,PC |
| Ribeiro et al. 2019 | Determinants of undesirable stable behaviour | TA | Past housed for >30 days Sampled 5 consecutive days 24hrs | 105 | Average 6-9yrs | M,G,S | Activity patterns including feeding, normal and undesirable behaviour | E3c | PHYS, PS | HR,RR,RT |
| Ruet et al. 2019 | Features of stable | TA | Long term scan sampling 50 days over 9 mnths | 187 | 4-20yrs | M,G,S | Stereotypical behaviour, aggression to humans, withdrawn / alert postures | E3a | PS,  -veSOC |  |
| Sauveroche et al. 2020 | Personality, management | TA | Long term / current housing  2 non-consec. days sampling | 153 | 3-28yrs | M,G | Social, feeding, stereotypical locomotory behaviour | E3a | PHYS,  PS | HC |
| Sénèque et al. 2019 | Posture and behaviour | TA | Long term over 8 months | 85 | Adult | M,G | Stereotypical/abnormal repetitive behaviour &  posture | E3c  E3b | PS |  |
| Stomp et al. 2018a | Snorts, welfare state | TA | Focal sampling over 10 days | 48 | 4-22yrs | M,G | Snorts | E1 | PS  +ve / -veEXP |  |
| Suagee-Bedore et al. 2021 | Size of turnout area | RM | 1 x 1 hour session in 2 conditions | 12 | 4-19yrs | M,G | Stress behaviour, altruistic and agonistic (5 levels) behav | E3c | PS  +ve/-ve SOC  PHYS | PC |
| Sundman et al. 2022 | Forage provision in group housing (net hole size) | RM | 2 weeks prep. 1 week x3 conditions | 15 | 7-19yrs | M,G | Agonistic threats, aggression, avoidance | E3a | -veSOC  AVO  PS |  |
| Visser et al. 2008 | Individual/paired first time housing/ novel object test | BS | 12 weeks. Behav. 9-5 wk 1-3 (5 days/wk).  Novel object test wk 12. | 36 | 2yrs | M,G | Behaviour in housing  Response to novel object | E3a  E3a | PS  Weight gain  PHYS: HR,HRV | PC  HR, HRV |
| Werhahn et al. 2012 | Turnout singly, in pairs or not, ridden | RM | 6 weeks – 3x 2-week periods, record 3 consec. days at end 2^nd^ wk | 6 | 4-10yrs | M,G | Effect of turnout on behavioural states in situ and ridden behaviour | E3a  E2a | PHYS  PS | HRV |
| Yarnell et al. 2015 | Social housing, handling test | RM | 4 weeks – 4x 5 days in each cond. | 16 | 6-21yrs | M,G | Behavioural state in different housing and reaction to temperature sampling | E3c  E3a | PS  PHYS | FC, ET, RT |

**Table S1.1** **references:**

**Baumgartner M, Erhard MH and Zeitler-Feicht MH** 2023 Which animal-to-feeding-place ratio at time-controlled hay racks is animal appropriate? Preliminary analysis of stress responses of horses. *Frontiers in Veterinary Science* **9**. <https://doi.org/10.3389/fvets.2022.1005102>

**Burla JB, Rufener C, Bachmann I, Gygax L, Patt A and Hillmann E** 2017 Space allowance of the littered area affects lying behavior in group-housed horses. *Frontiers in Veterinary Science* **4:** 1–12. <https://doi.org/10.3389/fvets.2017.00023>

**Greening L, Downing J, Amiouny D, Lekang L and McBride S** 2021 The effect of altering routine husbandry factors on sleep duration and memory consolidation in the horse. *Applied Animal Behaviour Science* **236:** 105229. <https://doi.org/10.1016/j.applanim.2021.105229>

**Harewood EJ and McGowan CM** 2005 Behavioral and physiological responses to stabling in naive horses. *Journal of Equine Veterinary Science* ***25*:** 164–170. <https://doi.org/10.1016/j.jevs.2005.03.008>

**Hildebrandt F, Büttner K, Krieter J and Czycholl I** 2022 The Behavior of Horses Stabled in a Large Group at Essential Resources (Watering Point and Lying Halls). *Journal of Equine Veterinary Science* **118**: 104106. <https://doi.org/10.1016/j.jevs.2022.104106>

**Lansade L, Lemarchand J, Reigner F, Arnould C and Bertin A** 2022 Automatic brushes induce positive emotions and foster positive social interactions in group-housed horses. *Applied Animal Behaviour Science* **246**: 105538 <https://doi.org/10.1016/j.applanim.2021.105538>

**Lansade L, Valenchon M, Foury A, Neveux C, Cole SW, Layé S, Cardinaud B, Lévy F and Moisan MP** 2014 Behavioral and transcriptomic fingerprints of an enriched environment in horses (*Equus caballus*). *PLoS ONE* **9**: 1–19. <https://doi.org/10.1371/journal.pone.0114384>

**Lesimple C, Gautier E, Benhajali H, Rochais C, Lunel C, Bensaïd S, Khalloufi A, Henry S and Hausberger M** 2019 Stall architecture influences horses’ behaviour and the prevalence and type of stereotypies. *Applied Animal Behaviour Science* **219:** 104833. <https://doi.org/10.1016/j.applanim.2019.104833>

**Marliani G, Sprocatti I, Schiavoni G, Bellodi A and Accorsi PA** 2021 Evaluation of Horses’ Daytime Activity Budget in a Model of Ethological Stable: A Case Study in Italy. *Journal of Applied Animal Welfare Science* **24**: 200–213. <https://doi.org/10.1080/10888705.2020.1857252>

**Melvin MV, Costello E and Colpoys JD** 2020 Enclosed versus ring feeders: Effects of round-bale feeder type on horse behavior and welfare. *Journal of Veterinary Behavior* **39**: 41–46. <https://doi.org/10.1016/j.jveb.2020.07.004>

**Pessoa GO, Trigo P, Mesquita Neto FD, Lacreta Junior ACC, Sousa TM, Muniz JA and Moura RS** 2016 Comparative well-being of horses kept under total or partial confinement prior to employment for mounted patrols. *Applied Animal Behaviour Science* **184:** 51–58. <https://doi.org/10.1016/j.applanim.2016.08.014>

**Ribeiro LB, Matzkeit TV, Nicolau JTde S, Castilha LD, de Oliveira FCL and Bankuti FI** 2019 Determinants of Undesirable Behaviors in American Quarter Horses Housed in Box Stalls. *Journal of Equine Veterinary Science* **80:** 69–75. <https://doi.org/10.1016/j.jevs.2019.07.005>

**Ruet A, Lemarchand J, Parias C, Mach N, Moisan MP, Foury A, Briant C and Lansade L** 2019 Housing horses in individual boxes is a challenge with regard to welfare. *Animals* ***9:*** 1–19. <https://doi.org/10.3390/ani9090621>

**Sauveroche M, Henriksson J, Theodorsson E, Svensson Holm AC and Roth LSV** 2020 Hair cortisol in horses (*Equus caballus*) in relation to management regimes, personality, and breed. *Journal of Veterinary Behavior* **37:** 1–7. <https://doi.org/10.1016/j.jveb.2019.12.002>

**Sénèque E, Lesimple C, Morisset S and Hausberger M** 2019 Could posture reflect welfare state? A study using geometric morphometrics in riding school horses. *PLoS ONE* **14:** 1–20. <https://doi.org/10.1371/journal.pone.0211852>

**Stomp M, Leroux M, Cellier M, Henry S, Lemasson A and Hausberger M** 2018a An unexpected acoustic indicator of positive emotions in horses. *PLoS ONE* **13(7):** 1–23. <https://doi.org/10.1371/journal.pone.0197898>

**Suagee-Bedore JK, Linden DR and Bennett-Wimbush K** 2021 Effect of Pen Size on Stress Responses of Stall-Housed Horses Receiving One Hour of Daily Turnout. *Journal of Equine Veterinary Science* **98**: 103366. <https://doi.org/10.1016/j.jevs.2020.103366>

**Sundman ER, Goodwin JD, Reisinger CN, Smith MR, Costello E, Walter K and Colpoys JD** 2022 Short Communication: Round bale hay net effects on agonistic behaviors of group fed horses. *Journal of Veterinary Behavior* **55–56**: 58–62. <https://doi.org/10.1016/j.jveb.2022.07.015>

**Visser EK, Ellis AD and Van Reenen CG** 2008 The effect of two different housing conditions on the welfare of young horses stabled for the first time. *Applied Animal Behaviour Science* ***114*(3–4):** 521–533. <https://doi.org/10.1016/j.applanim.2008.03.003>

**Werhahn H, Hessel EF and Van den Weghe HFA** 2012 Competition Horses Housed in Single Stalls (II): Effects of Free Exercise on the Behavior in the Stable, the Behavior during Training, and the Degree of Stress. *Journal of Equine Veterinary Science* ***32*(1):** 22–31. <https://doi.org/10.1016/j.jevs.2011.06.009>

**Yarnell K, Hall C, Royle C and Walker SL** 2015 Domesticated horses differ in their behavioural and physiological responses to isolated and group housing. *Physiology and Behavior* ***143:*** 51–57. <https://doi.org/10.1016/j.physbeh.2015.02.040>

**Table S1.2:** **Study details for the 20 retained articles relating to intra-species social behaviour**

| **Study** | **Focus of study and**  **other tests included** | **Study design** | **Length of study** | **Subject details** | | | **Behaviour recorded** | **Type of ethogram used]** | **Supporting evidence** | **Physiological measures** |
| --- | --- | --- | --- | --- | --- | --- | --- | --- | --- | --- |
|  |  |  |  | **No.** | **Age** | **Sex** |  |  |  |  |
| Briefer et al. 2015 | Arousal/valence in whinnies | TA | 4 days (day 1 habituation) | 20 | 6-31yrs | M,G | Whinnies, head movement, position, locomotion, chewing | E1 | PS, PHYS | HR, HRV, RR,ST |
| Briefer et al. 2017 | Recognition of valency, familiarity of whinnies | TA | 2 days / 2 treatments | 18 | Unspecified | M,G | Whinnies, head movement, position, locomotion, chewing | E1, E2c | PS | HRV,RR,ST |
| Christensen et al. 2011 | Group changes | RM,OB | 7 weeks, group member changed each week | 45 | 2yrs | M | +ve and -ve social interactions, play | E3b, E3c | PS,  +ve / -ve SOC |  |
| da Cruz et al. 2023 | Laterality of social and resting behaviour in feral horses | OB | 2 months | 37 | Various Not known | M,S,C,F | Recumbent resting, cross-resting, grooming, displacement & threats | E3c | PS, +ve / -ve SOC |  |
| Farmer et al. 2018 | Laterality of affiliative interactions | OB | 17 days | 31 | Foal-20yrs | M,S,G, F,C | Affiliative & non-affiliativ. approaches / interactions | E3b, E3c | PS, +ve / -ve SOC |  |
| Górecka-Bruzda et al. 2016 | Yawning | OB | 4-10hrs per subject over 2 months | 35 | 1yr-adult | M,S,G, F,C | Social interactions  Yawning | E3c  E1 | PS, +ve / -ve SOC |  |
| Górecka-Bruzda et al. 2022 | Choice test: Food / equine companion | BS | Two tests, two-week gap then third test | 24 | 9-20yrs | M,G | Behaviour indicative of separation anxiety | E3c |  |  |
| Hausberger et al. 2012 | Welfare and adults’ play behaviour | OB | 2 x 1hr session over 1 month (gap of 1-2 wks) | 29 | 7-17yrs | M,G | Social play behaviour | E3c | PS, PIP | PC |
| Kieson et al. 2023 | Allogrooming | OB | 3 months – approx..40 hrs video | 200 | 6-20yrs | M | Allo-grooming | E3c | PS, +ve SOC |  |
| Maigrot et al. 2017 | Valence in whinnies (Przewalski horses) | OB / record audio | Opportunistic sampling | 23 | Varied | Not spec. | Type of vocalisation | E1 | PS, +ve / -ve SIT; +ve /-ve SOC |  |
| Maigrot et al. 2022 | Cross species recognition of vocal valence | OB / record audio | 5-month period | 24 | Adult | Not spec. | Posture and locomotion response | E2b | PS,  +ve/-ve SIT |  |
| Majecka & Klawe 2018 | Paddock size and social interactions | OB | 4-5 mnths, 2 mnths per condition | 78 | 3mnths-30yrs | M,S,G, F,C | 7 categories interaction: 4 aggressive, 3 friendly | E3c | PS, +ve / -ve SOC |  |
| Nuñez et al. 2014 | Group changes in feral mares | OB | 3 mnths in breeding season, 1 mnth non-br | 24 | Adult | M | Group changes in mares (contraception effect) |  | PS, PHYS | FC |
| Pierard et al. 2019 | Group density and interactions | OB | 2 mnths | 12 | 1-29yrs | M,G | Affiliative / agonistic interactions | E3c | PS, +ve / -ve SOC |  |
| Pond et al. 2010 | Characterisation of vocalisation | OB / record audio | Not specified | 27 | Adult & foals | M,F,C | Vocalisation +ve / -ve situs | E1 | PS, +ve / -ve SIT |  |
| Stachurska et al. 2023 | Oestrus / dioestrus behaviour | RM,TA OB | 6 successive oestrus days, every 3^rd^ day dioestrus =5 | 15 | 7-10yrs | M | Affiliative / agonistic interactions, activity | E3c | PS, PHYS, +ve -ve SOC | HR,HRV,RT, ST |
| Stomp et al. 2018b | Snorts | TA,OB | 10 days | 20 | 4-15yrs | M,G | Snorts in diff locations, ear position | E2c | PS,  +ve -ve SIT |  |
| Strand et al. 2002 | Isolation from group | BS | 6 days (baseline + 5 indiv. test days) | 15 | Adult | M | Activity, social interactions | E3b | PS, PHYS (HR), -ve SOC | HR,PC |
| Wathan et al. 2016 | 2D horse facial expression images | Choice test | 2 trials per horse | 48 | 3-32yrs | M,G | Response to 2D facial images of con specifics | E3c | PS, PHYS, APP | HR |
| York & Schulte 2014 | Behaviour of lactating / non-lactating mares | BS,OB | 3 months | 27 | 6-21yrs | M | Activity, feeding, socialisation | E3c | N/A | FC |

**Table S1.2 references:**

**Briefer EF, Maigrot AL, Mandel R, Freymond SB, Bachmann I and Hillmann E** 2015 Segregation of information about emotional arousal and valence in horse whinnies. *Scientific Reports* **4**: 1–11. <https://doi.org/10.1038/srep09989>

**Briefer EF, Mandel R, Maigrot AL, Briefer Freymond S, Bachmann I and Hillmann E** 2017 Perception of emotional valence in horse whinnies. *Frontiers in Zoology* 14: 1–12. <https://doi.org/10.1186/s12983-017-0193-1>

**Christensen JW, Søndergaard E, Thodberg K and Halekoh U** 2011 Effects of repeated regrouping on horse behaviour and injuries. *Applied Animal Behaviour Science* **133:** 199–206. <https://doi.org/10.1016/j.applanim.2011.05.013>

**da Cruz AB, Hirata S, dos Santos ME and Mendonça RS** 2023 Show me your best side: Lateralization of social and resting behaviors in feral horses. *Behavioural Processes* **206**: February. <https://doi.org/10.1016/j.beproc.2023.104839>

**Farmer K, Krüger K, Byrne RW and Marr I** 2018 Sensory laterality in affiliative interactions in domestic horses and ponies (*Equus caballus*). *Animal Cognition* **21**: 631–637. <https://doi.org/10.1007/s10071-018-1196-9>

**Górecka-Bruzda A, Fureix C, Ouvrard A, Bourjade M and Hausberger M** 2016 Investigating determinants of yawning in the domestic (*Equus caballus*) and Przewalski (*Equus ferus* przewalskii) horses. *Science of Nature* **103**: 9–10. <https://doi.org/10.1007/s00114-016-1395-7>

**Górecka-Bruzda A, Jastrzębska E, Drewka M, Nadolna Z, Becker K and Lansade L** 2022 Female horses are more socially dependent than geldings kept in riding clubs. *Applied Animal Behaviour Science* **254**: July. <https://doi.org/10.1016/j.applanim.2022.105714>

**Hausberger M, Fureix C, Bourjade M, Wessel-Robert S and Richard-Yris MA** 2012 On the significance of adult play: What does social play tell us about adult horse welfare? *Naturwissenschaften* **99**: 291–302. <https://doi.org/10.1007/s00114-012-0902-8>

**Kieson E, Goma AA and Radi M** 2023 Tend and Befriend in Horses: Partner Preferences, Lateralization, and Contextualization of Allogrooming in Two Socially Stable Herds of Quarter Horse Mares. *Animals* **13**: 225. <https://doi.org/10.3390/ani13020225>

**Maigrot AL, Hillmann E, Anne C and Briefer EF** 2017 Vocal expression of emotional valence in Przewalski’s horses (*Equus przewalskii*). *Scientific Reports* **7**: 1–11. <https://doi.org/10.1038/s41598-017-09437-1>

**Maigrot AL, Hillmann E and Briefer EF** 2022 Cross-species discrimination of vocal expression of emotional valence by Equidae and Suidae. *BMC Biology* **20**: 1–14. <https://doi.org/10.1186/s12915-022-01311-5>

**Majecka K and Klawe A** 2018 Influence of Paddock Size on Social Relationships in Domestic Horses. *Journal of Applied Animal Welfare Science* **21**: 8–16. <https://doi.org/10.1080/10888705.2017.1360773>

**Nuñez CMV, Adelman JS, Smith J, Gesquiere LR and Rubenstein DI** 2014 Linking social environment and stress physiology in feral mares (*Equus caballus*): Group transfers elevate fecal cortisol levels. *General and Comparative Endocrinology* **196**: 26–33. <https://doi.org/10.1016/j.ygcen.2013.11.012>

**Pierard M, McGreevy P and Geers R** 2019 Effect of density and relative aggressiveness on agonistic and affiliative interactions in a newly formed group of horses. *Journal of Veterinary Behavior* **29**: 61–69. <https://doi.org/10.1016/j.jveb.2018.03.008>

**Pond RL, Darre MJ, Scheifele PM and Browning DG** 2010 Characterization of equine vocalization. *Journal of Veterinary Behavior: Clinical Applications and Research* **5**: 7–12. <https://doi.org/10.1016/j.jveb.2009.08.002>

**Stachurska A, Kędzierski W, Kaczmarek B, Wiśniewska A, Żylińska B and Janczarek I** 2023 Variation of Physiological and Behavioural Parameters during the Oestrous Cycle in Mares. *Animals* **13(2)**: 211. <https://doi.org/10.3390/ani13020211>

**Stomp M, Leroux M, Cellier M, Henry S, Hausberger M and Lemasson A** 2018b Snort acoustic structure codes for positive emotions in horses. *Science of Nature* **105**: 9–10. <https://doi.org/10.1007/s00114-018-1582-9>

**Strand SC, Tiefenbacher S, Haskell M, Hosmer T, McDonnell SM and Freeman DA** 2002 Behavior and physiologic responses of mares to short-term isolation. *Applied Animal Behaviour Science* **78**: 145–157. <https://doi.org/10.1016/S0168-1591(02)00106-5>

**Wathan J, Proops L, Grounds K and McComb K** 2016 Horses discriminate between facial expressions of conspecifics. *Scientific Reports* **6:** 1–11. <https://doi.org/10.1038/srep38322>

**York CA and Schulte BA** 2014 The relationship of dominance, reproductive state and stress in female horses (*Equus caballus*). *Behavioural Processes* **107:** 15–21. <https://doi.org/10.1016/j.beproc.2014.07.005>

**Table S1.3*:***  **Study details for the 14 retained articles relating to the behaviour of the mare and foal during weaning**

| **Study** | **Focus of study &**  **other tests included** | **Study design** | **Length of study** | **Subject details** | | | **Behaviour recorded** | **Type of ethogram used** | **Supporting evidence** | **Physiological measures** |
| --- | --- | --- | --- | --- | --- | --- | --- | --- | --- | --- |
|  |  |  |  | **No.** | **Age** | **Sex** |  |  |  |  |
| Delank et al. 2023 | Weaning stress | OB | 8hrs day before, day after, 4,8,18- 20 days post. | 10 | Foals <1yr | F,C | Time budget  Interactions inc. play | E3c | PS, PHYS | FC |
| Erber et al. 2012 | Weaning protocol | BS | 2hrs am+pm, 1 day before and 8 days after weaning | 17 | Foals <1yr | F,C | Vocalisation, defecation, feeding. Activity, lying locomotion | E2a | PS, PHYS (SC) | HRV,SC |
| Falomo et al. 2020 | Effect of weaning protocol on mares | BS | 7 days before weaning, day of weaning, 7, 30 days post-w. | 22 | 7-20yrs | M | Stress behaviours (inc. -ve SOC) | E3c | PS, PHYS (SC) | SC,HC |
| Górecka-Bruzda et al. 2015 | Weaning semi-feral vs stabled foals | BS | 6hrs weaning day & 6hrs 1 day post w. | 53 | 7-9 mnths | F,C | Activity and maintenance behaviours | E3c | PS, PHYS | FC |
| Henry et al. 2012 | Adult company at weaning | BS | Twice on day before, day of, and day after wean. | 32 | 4.5-7 mnths | F,C | Activity, rest, interactions, suckling, events | E3c | PS, PHYS | SC |
| Hoffman et al. 1995 | Diet, individual or paired housing | BS | 2 weeks starting from weaning - 4 days/30mins | 18 | 5-7 mnths | F,C | Scoring:  Eustress, mild distress, severe distress | E2c | PS, PHYS | PC |
| Lansade et al. 2018 | Sudden or progressive weaning | BS | 4 weeks pre-weaning, day of weaning, 3 months post weaning | 34 x 2 mare foal | Adult, average 7 mnths | M,F,C | Activity, social interaction, stress, pen location Personality | E3a | PS, PHYS | SC |
| Merkies et al. 2016 | Two-stage weaning | BS | 4 days pre-treatment, 4 days treatment, 4 days post w. | 15 x 2 mare & foal | Average 9.8yrs, 5.5 mnths | M,F,C | Activity, aggression, drinking, nursing | E3a | PS, PHYS (FC) | HR,FC |
| Moons et al. 2005 | Mare-foal separation pre-weaning | BS | 10 mins separations from 2 weeks old (2,4,6,8,10, 12 wks). Test 3 days (pre-wean, wean and post-w) | 10 | Average 6 mnths | F,C | Mare-maternal care post ST separation. Foals at w. activity, vocalisation, defecation, pawing. | E3a  E2a | PS, PHYS | HR,SC |
| Nicol et al. 2005 | Diet, weaning method, temperament tests | BS | Foals OB at 2-40 wks old, 3x 15 mins OB at weaning, reactivity tested age 9-22mnths | 17 | 2-40 weeks | F,C | Time budgets, interactions.  Reactivity tests. | E3a | PS |  |
| Pérez Manrique et al. 2019 | Individual differences (separation pre-weaning) | TA,RM | 4 x short (10min) separation when foals 1 wk post-natal to 6 months | 30 | 1 week – 6 mnths | F,C | Alertness  Vocalisations Tail raised Head tosses Locomotion.  Maternal vocalisations | E3c | PS, PHYS | HRV |
| Rogers et al. 2012 | Foal image during separation from foal | RM | 10mins x 3 conditions at foal ages 4,15, 30, 45, 60 days | 7 | Average 10yrs | M | Level of agitation | E3c | PS, PHYS | HR,PC |
| Waters et al. 2002 | Development of stereotypical behaviour | TA | Long term followed up to 4 years – Obs. Then questionnaire. | 225 | NA | M,G,S,F,C | Ranking (threats given/ received).  Abnormal behaviour | E2a | PS | NA |
| Wulf et al. 2018 | Effect of sex of foal on response to abrupt weaning | BS | 1hr am/pm 2 days pre – 7 days post w, 4 consec. hrs at weaning | 22 | 6 mnths | F,C | Vocalisation, lying, defecation, feeding. | E2b | PS, PHYS | HR,HRV,SC |

**Table S1.3 references:**

**Delank K, Reese S, Erhard M and Wöhr AC** 2023 Behavioral and hormonal assessment of stress in foals (*Equus caballus*) throughout the weaning process*. PLoS ONE* **18**(: 1–16. <https://doi.org/10.1371/journal.pone.0280078>

**Erber R, Wulf M, Rose-Meierhöfer S, Becker-Birck M, Möstl E, Aurich J, Hoffmann G and Aurich C** 2012 Behavioral and physiological responses of young horses to different weaning protocols: A pilot study. *Stress* **15**: 184–194. <https://doi.org/10.3109/10253890.2011.606855>

**Falomo ME, Gabai G, Franchini G, Poltronieri C, Rossi M and Normando S** 2020 Behavioral and hormonal effects of 2 weaning methods in trotter mares*. Journal of Veterinary Behavior* **35**: 47–53. <https://doi.org/10.1016/j.jveb.2019.10.005>

**Górecka-Bruzda A, Suwała M, Palme R, Jaworski Z, Jastrzebska E, Boroń M and Jezierski T** 2015 Events around weaning in semi-feral and stable-reared Konik polski foals: Evaluation of short-term physiological and behavioural responses. *Applied Animal Behaviour Science* **163:** 122–134. <https://doi.org/10.1016/j.applanim.2014.11.004>

**Henry S, Zanella AJ, Sankey C, Richard-Yris MA, Marko A and Hausberger M** 2012 Adults may be used to alleviate weaning stress in domestic foals (Equus caballus). *Physiology and Behavior* **106(4)**: 428–438. <https://doi.org/10.1016/j.physbeh.2012.02.025>

**Hoffman RM, Kronfel, DS, Holland JL and Greiwe-Crandell KM** 1995 Preweaning diet and stall weaning method influences on stress response in foals. *Journal of Animal Science* **73(10):** 2922–2930. <https://doi.org/10.2527/1995.73102922x>

**Lansade L, Foury A, Reigner F, Vidament M, Guettier E, Bouvet G, Soulet D, Parias C, Ruet A, Mach N, Lévy F and Moisan MP** 2018 Progressive habituation to separation alleviates the negative effects of weaning in the mother and foal. *Psychoneuroendocrinology* **97**: 59–68. <https://doi.org/10.1016/j.psyneuen.2018.07.005>

**Merkies K, DuBois C, Marshall K, Parois S, Graham L and Haley D** 2016 A two-stage method to approach weaning stress in horses using a physical barrier to prevent nursing. *Applied Animal Behaviour Science* **183:** 68–76. <https://doi.org/10.1016/j.applanim.2016.07.004>

**Moons CPH, Laughlin K and Zanella AJ** 2005 Effects of short-term maternal separations on weaning stress in foals. *Applied Animal Behaviour Science* **91**: 321–335. <https://doi.org/10.1016/j.applanim.2004.10.007>

**Nicol CJ, Badnell-Waters AJ, Bice R, Kelland A, Wilson AD and Harris PA** 2005 The effects of diet and weaning method on the behaviour of young horses. *Applied Animal Behaviour Science* **95**: 205–221. <https://doi.org/10.1016/j.applanim.2005.05.004>

**Pérez Manrique L, Hudson R, Bánszegi O and Szenczi P** 2019 Individual differences in behavior and heart rate variability across the preweaning period in the domestic horse in response to an ecologically relevant stressor. *Physiology and Behavior* **210:** 112652. <https://doi.org/10.1016/j.physbeh.2019.112652>

**Rogers CW, Walsh V, Gee EK and Firth EC** 2012 A preliminary investigation of the use of a foal image to reduce mare stress during mare-foal separation. *Journal of Veterinary Behavior: Clinical Applications and Research* **7**: 49–54. <https://doi.org/10.1016/j.jveb.2011.04.006>

**Waters AJ, Nicol CJ and French NP** 2002 Factors influencing the development of stereotypic and redirected behaviours in young horses: Findings of a four year prospective epidemiological study. *Equine Veterinary Journal* **34(6):** 572–579. <https://doi.org/10.2746/042516402776180241>

**Wulf M, Beythien E, Ille N, Aurich J and Aurich C** 2018 The stress response of 6-month-old horses to abrupt weaning is influenced by their sex. *Journal of Veterinary Behavior: Clinical Applications and Research* **23**: 19–24. <https://doi.org/10.1016/j.jveb.2017.10.010>

**Table S1.4: Study details for the 8 retained articles relating to experimental tests of affective state (judgement bias testing)**

| **Study** | **Focus of study &**  **other tests included** | **Study design** | **Length of study** | **Subject details** | | | **Behaviour recorded** | **Type of ethogram used** | **Supporting evidence** | **Physiological measures** |
| --- | --- | --- | --- | --- | --- | --- | --- | --- | --- | --- |
|  |  |  |  | **No.** | **Age** | **Sex** |  |  |  |  |
| Briefer Freymond et al. 2014 | Effect of +ve/-ve reinforcement on mood | BS | 12 days trials in 5 phases: 3 days training, 2 days testing | 12 | 9-20yrs | M | +ve / -ve signs during training  Testing | E2c | +ve bias |  |
| Henry et al. 2017 | Effect of home environment | BS | Training 5 consec. 180s, testing 9x 180s (+ve,-ve, ambig.) | 34 | 10-25yrs | M,G,S | Chronic welfare score followed by testing | E2b | Judgement bias associated with welfare |  |
| Hintze & Schanz 2021 | Eye wrinkles and optimism | TA | Training to criterion, 6 test sessions | 16 | 4-22yrs | S | Eye wrinkles / bias | E3c | Inconclusive (association with eye wrinkles) |  |
| Löckener et al. 2016 | Change for better (home environment) | RM | Housing 6 mnths, 10 days pasture. Training 10 sessions, test 9 trials | 13 | 6-22yrs | M,G | Judgement bias | None cited | +ve Bias |  |
| Marliani et al. 2022a | Type of home environment | BS | Habituation, 2-5 training sessions, test on 2 consec. days | 41 | Average 14-18yrs | M,G,S | BCS food motivation  Personality | E2c | Inconclusive | FC,HC |
| Marliani et al. 2022b | Individual differences, personality | TA | Habituation, training, test on 2 consec. days | 16 | 5-25yrs | M,G,S | Personality  Stress in training / testing | E2c  E3c | Individual differences found | FC,HC |
| Marr et al. 2018 | Motor and sensory laterality and optimism | TA | Habituation, training, testing (one ambig.) | 17 | 3-26yrs | M,G | Motor and sensory laterality | E2c | Association laterality / cog. bias |  |
| McGuire et al. 2018 | Rescued from neglect vs non-rescued | BS | Habituation 3+ trials, training 6 trials per day – 4 days. Test 2 consec. days | 8 and 2 donkeys | 8-24yrs (horses) | M,G | Judgement bias |  | +ve bias |  |

**Table S1.4 references:**

**Briefer Freymond S, Briefer EF, Zollinger A, Gindrat-von Allmen Y, Wyss C and Bachmann I** 2014 Behaviour of horses in a judgment bias test associated with positive or negative reinforcement. *Applied Animal Behaviour Science* **158:** 34–45. <https://doi.org/10.1016/j.applanim.2014.06.006>

**Henry S, Fureix C, Rowberry R, Bateson M and Hausberger M** 2017 Do horses with poor welfare show ‘pessimistic’ cognitive biases? *Science of Nature* **104**: 1–2. <https://doi.org/10.1007/s00114-016-1429-1>

**Hintze S and Schanz L** 2021 Using the Judgment Bias Task to Identify Behavioral Indicators of Affective State: Do Eye Wrinkles in Horses Reflect Mood? *Frontiers in Veterinary Science* **8(July)**: 1–6. <https://doi.org/10.3389/fvets.2021.676888>

**Löckener S, Reese S, Erhard M and Wöhr AC** 2016 Pasturing in herds after housing in horseboxes induces a positive cognitive bias in horses. *Journal of Veterinary Behavior: Clinical Applications and Research* **11**: 50–55. <https://doi.org/10.1016/j.jveb.2015.11.005>

**Marliani G, Balboni A, Tiberi C, Malavasi R, Gardini A and Accorsi PA** 2022a Is the judgment bias test a good tool to assess the quality of horse management? *Journal of Veterinary Behavior* **58:** 62–69. <https://doi.org/10.1016/j.jveb.2022.11.002>

**Marliani G, Vannucchi I, Kiumurgis I and Accorsi PA** 2022b Limitations of Spatial Judgment Bias Test Application in Horses (*Equus ferus caballus*). *Animals* **12**: (21). <https://doi.org/10.3390/ani12213014>

**Marr I, Farmer K and Krüger K** 2018 Evidence for right-sided horses being more optimistic than left-sided horses. *Animals* **8**: (12). <https://doi.org/10.3390/ani8120219>

**McGuire MC, Johnson-Ulrich Z, Robeson A, Zeigler-Hill V and Vonk J** 2018 I say thee “neigh”: Rescued equids are optimistic in a judgment bias test. *Journal of Veterinary Behavior* **25**: 85–91. <https://doi.org/10.1016/j.jveb.2018.03.009>

**Table S1.5: Study details for the 7 retained articles relating to stereotypical behaviour**

| **Study** | **Associated behavioural characteristic** | **Study design** | **Length of study** | **Subject details** | | | **Behaviour recorded** | **Type of ethogram used** | **Supporting evidence** | **Physiological measures** |
| --- | --- | --- | --- | --- | --- | --- | --- | --- | --- | --- |
|  |  |  |  | **No.** | **Age** | **Sex** |  |  |  |  |
| Arena et al. 2021 | Time in stable, work frequency | TA | Long term effects BS (no time scale) | 54 | Average 11yrs | M,G,S | Abnormal / normal behaviour groups | NA | PHYS | PC, HC |
| Briefer Freymond et al. 2020 | Stereotypies and learning, coping | BS | 6 spatial learning trials in 1 day | 37 | 3-24yrs | M,G,S | Learning performance Stress – attention / frustration | E3c | PS,PHYS  Learning performance | HR,HRV,SC |
| Briefer Freymond et al. 2019 | Stereotypies and learning | BS | Acclimation 2-6 days, training 3-7 weeks | 13 | 10-25yrs | M,G,S | Learning performance  Crib biting | E3c | PS, learning performance | HR,HRV |
| Freymond et al. 2019 | Stereotypies and tactile sensitivity | BS | 5 tests per horse in 1 day | 37 | 4-24yrs | M,G,S | Response to ‘personality’ tests | E3c | PS, AVO |  |
| Hanis et al. 2021 | Type of work and diet | OB,TA | 3 days scan s every 10mins for 12hrs | 207 | 7-25yrs | Not given | Oral SB / redirected behaviour | E3c | Association diet and oral behaviour |  |
| Hausberger et al. 2009 | Type of work (and stereotypies) | TA | 4x scan sampling every 10secs for 5 mins - 3 sessions/day | 76 | 6-15yrs | G | Stereotypical behaviour | E3c | PS |  |
| Zuluaga et al. 2018 | Type of work | OB | 48hrs continuous recording | 20 | Average 12yrs | G | Normal, abnormal & stereotypical behaviour | E2c | PS (cortisol index didn’t relate to behaviour) | PC |

**Table S1.5 references:**

**Arena I, Marliani G, Sabioni S, Gabai G, Bucci D and Accorsi PA** 2021 Assessment of horses’ welfare: Behavioral, hormonal, and husbandry aspects. *Journal of Veterinary Behavior* **41**: 82–90. <https://doi.org/10.1016/j.jveb.2021.01.006>

**Briefer Freymond S, Beuret S, Ruet A, Zuberbühler K, Bachmann I and Briefer EF** 2020 Stereotypic behaviour in horses lowers stress but not spatial learning performance. *Applied Animal Behaviour Science* **232**: 105099. <https://doi.org/10.1016/j.applanim.2020.105099>

**Briefer Freymond S, Ruet A, Grivaz M, Fuentes C, Zuberbühler K, Bachmann I and Briefer EF** 2019 Stereotypic horses (Equus caballus) are not cognitively impaired. *Animal Cognition* **22:** 17–33. <https://doi.org/10.1007/s10071-018-1217-8>

**Freymond SB, Bardou D, Beuret S, Bachmann I, Zuberbühler K and Briefer EF** 2019 Elevated sensitivity to tactile stimuli in stereotypic horses. *Frontiers in Veterinary Science* **6:** MAY. <https://doi.org/10.3389/fvets.2019.00162>

**Hanis F, Chung ELT, Kamalludin MH and Idrus Z** 2021 Do nutrient composition of feedstuffs affect the proportion of oral stereotypies and redirected behaviors among horse working groups? *Journal of Veterinary Behavior* **46**: 7–14. <https://doi.org/10.1016/j.jveb.2021.07.010>

**Hausberger M, Gautier E, Biquand V, Lunel C and Jégo P** 2009 Could work be a source of behavioural disorders? A study in horses*. PLoS ONE* **4:** 2–8. <https://doi.org/10.1371/journal.pone.0007625>

**Zuluaga AM, Mira A, Sánchez JL and Martínez AJR** 2018 Frequency of abnormal and stereotypic behaviors in urban police patrolling horses: A continuous 48-hour study. *Revista Colombiana de Ciencias Pecuarias* **31(1):** 17–25. <https://doi.org/10.17533/udea.rccp.v31n1a03>
